# Decrypting the Unusual Structure and σ-Hole Interactions of the XC(NO_2_)_3_ (X=F, Cl, Br, and I) Compounds Using Quasi-Atomic Orbitals

**DOI:** 10.3390/molecules30091986

**Published:** 2025-04-29

**Authors:** Emilie B. Guidez

**Affiliations:** Department of Chemistry, University of Colorado Denver, Denver, CO 80204, USA; emilie.guidez@ucdenver.edu

**Keywords:** halogen bonding, kinetic energy, quasi-atomic orbitals, sigma-hole interactions

## Abstract

This work reports the quasi-atomic orbital analysis of the XC(NO_2_)_3_ (X=F, Cl, Br, and I) compounds and shows that the interactions between the C-N σ bonds and the lone electron pairs on the halogen atom and oxygen atoms of the nitro groups may contribute to the unusually short C-X distances observed. While the presence of a σ-hole on the halogen atom of the XC(NO_2_)_3_ compound may not be obvious from the electron density distribution, an analysis of the intermolecular forces of the NH_3_--XC(NO_2_)_3_ complexes suggests a σ -hole interaction between the nitrogen lone pair and halogen atom X (X=Cl, Br, and I) in the linear N--X-C configuration, where electrostatics and exchange forces dominate. The linear N--X-C bond in these systems is shown to have a noticeable covalent character, which is captured in the polarization energy term. Complexation with the ammonia nucleophile is shown to affect the electronic structure of the entire compounds, notably the oxygen/halogen lone electron pairs interactions with the C-N σ bonds.

## 1. Introduction

A σ-hole corresponds to a localized region of positive electrostatic potential on a covalently bonded halogen, chalcogen, or pnictogen atom in the third row of the periodic table or lower [1,2,3,4]. Accordingly, σ-hole interactions are defined as a family of noncovalent forces between a σ-hole of a molecule and a negative site, such as a nucleophile. The strength and directionality of these interactions can be tuned by modifying the electron-donating capacity of the nucleophile, the atom containing the σ-hole(s), or by modifying the electron-withdrawing (or donating) capacity of the covalently bonded substituents to that atom [3,5,6,7,8]. The magnitude of these interactions can compete with that of hydrogen bonds [9], offering new possibilities in the synthesis of molecular assemblies. In fact, in the past two decades, σ-hole interactions have been exploited to build new solid materials with desirable structural, chemical, and physical properties [10] via co-crystallization. [2,11] Their importance has also been revealed in other fields such as biology (DNA junctions [12], membrane transport [13], and protein–ligand interactions [14,15]), molecular recognition [16], and drug discovery [17,18].

**Figure 1:**
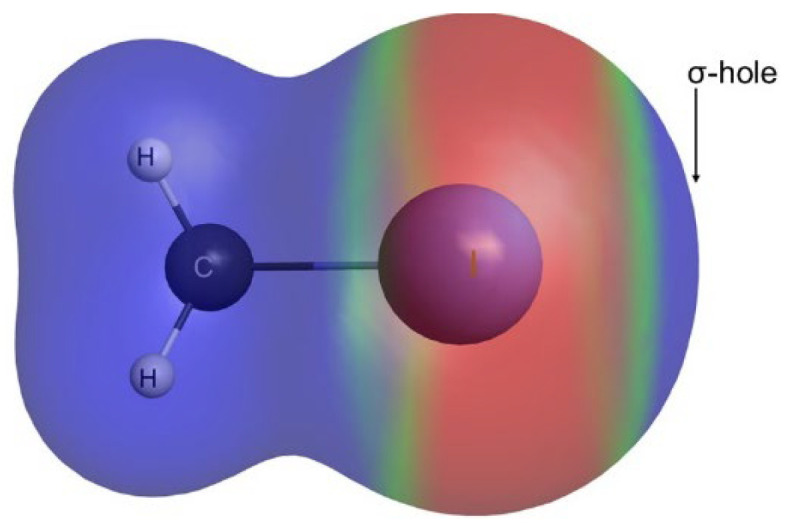
Molecular electrostatic potential map of CH_3_I. Blue indicates a region of positive electrostatic potential and red indicates a region of negative electrostatic potential.

The most widely studied type of σ-hole interaction is the halogen bond [2,19,20,21], where a single σ-hole is present on the extension of a covalently bonded halogen atom, while the remainder of the halogen surface (the belt) shows a negative electrostatic potential (Figure 1). Several studies have shown that, in addition to electrostatics, charge transfer [22,23], polarization [24], repulsion [25], and even dispersion forces [26,27] all play an important role and must be considered to provide an accurate picture of σ-hole interactions [28,29]. Several publications have also revealed that these interactions can possess a significant covalent character [30,31,32,33].

**Figure 2:**
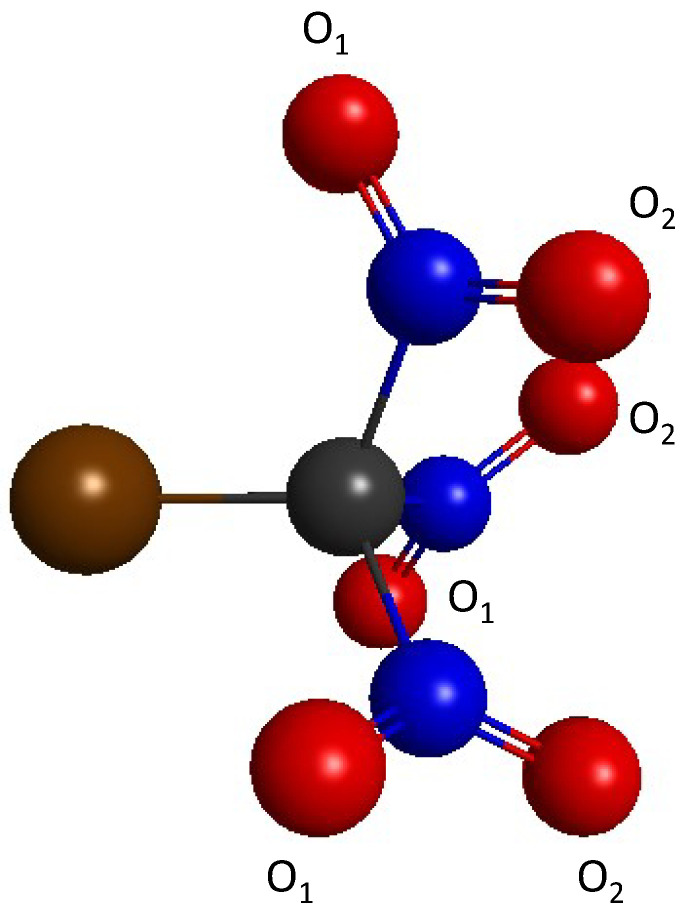
Structure of the XC(NO_2_)_3_ compound (X=F, Cl, Br, and I). Color coding: Brown = X, Black = C, Blue = N, and Red = O.

Electron density maps and electrostatic potential calculations have been extensively utilized to visualize σ-holes and predict how they may interact with other molecules [3,5,7,34,35]. A study by Klapötke et al. [36] reveals that, in the halotrinitromethane XC(NO_2_)_3_ compounds (X=F, Cl, Br, and I) presented in Figure 2, the electrostatic potential on the entire surface area of the halogen atom (including for X=F) is positive. Furthermore, the experimental C-Cl bond length of ClC(NO_2_)_3_ is 1.694 Å in the solid-state [36] and 1.712 Å in the gas phase [37,38], corresponding to the shortest C-Cl bond ever obtained for a tetrahedral carbon compound. Interestingly, such short C-Cl bonds do not occur in compounds with other strong electron-withdrawing groups such as ClC(CN)_3_ (R_C-Cl_ = 1.781 Å [39]) or ClCF_3_ (R_C-Cl_ = 1.752 Å [40]). The unique electronic and geometrical properties of these XC(NO_2_)_3_ propeller systems (the three nitro groups form a propeller-shaped structure) have been studied both experimentally [37,38,41] and computationally [35,42,43]. These studies suggest that intramolecular interactions between oxygen atoms of the nitro groups (labeled O_1_ in Figure 2) and the positively charged halogen (X=Cl, Br, and I) atom are at the origin of the short C-X bond length.

In this work, the bonding patterns in the XC(NO_2_)_3_ compound are elucidated using the quasi-atomic orbital (QUAO) analysis by Ruedenberg et al., [44,45] which provides valuable insight into the important role of the kinetic energy in driving covalent bonding. It is noted that this QUAO scheme has been used to elucidate ambiguous bonding patterns in several systems such as disilene molecules [46], agostic bonds [47], hydrogen-bonded complexes [48], infinitene [49], and more [50,51]. The objective of this study is two-fold: (1) analyze the covalent interactions in the XC(NO_2_)_3_ compound (X=F, Cl, Br, and I) using the QUAO method and determine how they affect the molecular structure, notably the C-X bond length; and (2) elucidate the covalent and non-covalent interactions involved in the formation of the NH_3_--XC(NO_2_)_3_ complex, using the QUAO method and the energy decomposition analysis in terms of intermolecular forces (IMF) by Su and Li [52], respectively.

## 2. Evaluation of Covalent and Non-Covalent Interactions

### 2.1. QUAO Analysis

A QUAO analysis is performed to gain insight into the covalent character of the bonds formed between atoms. A brief description of the method is given here, with more details given elsewhere [44,45,53]. First, the QUAOs are built by projecting the Hartree–Fock molecular orbitals and valence virtual orbitals (VVOs) [54] onto an Accurate Atomic Minimal Basis set (AAMBS) [55,56], spanning the full atomic valence space. AAMBS are a minimal orthogonal basis set, independent of the basis used to construct the molecular orbitals, that provides a very close representation of the ground state multi-configurational wave function of the atom. QUAOs are then hybridized into oriented QUAOs [44,57], which will be simply referred to as QUAOs in the remainder of this manuscript. These oriented QUAOs are built so as to maximize the number of small off-diagonal density matrix elements [57]. Most oriented QUAOs localized on a given atom are oriented towards a single covalently bonded atom, therefore revealing the covalent interactions between these atoms. The convention to label QUAOs is as follows:

- The symbol of the atom the QUAO is localized on is indicated with an upper-case letter. The symbol of the atom it is oriented towards is indicated with a lower-case letter. The type of bonding interaction is given next. For instance, the label Cnσ refers to a QUAO localized on atom C oriented towards atom N, via a σ bond.
- For atoms containing *p* lone pairs, the symbol of the atom where the lone pair is located is followed by the label *lp*. It is noted that *s* lone pairs are also present on the oxygen and halogen atoms but, since they do not contribute to the bonding interactions in these systems, they are not discussed further.

Interactions between QUAOs are characterized with bond orders (BOs) and kinetic bond orders (KBOs). The bond orders $\rho_{Aa,Bb}$ between QUAOs *Aa* and *Bb* correspond to the off-diagonal elements of the first order density matrix $\rho \left(1,2\right)$ expressed in terms of QUAOs and given by [44]:

(1) $$\rho \left(1,2\right)=\sum_{Aa}\sum_{Bb}\left.\left|Aa(1)\right.\right\rangle \rho_{Aa,Bb}\left\langle \left.Bb(2)\right|\right.$$

In Equation (1), *Aa* refers to QUAO *a*, localized on atom *A*, and *Bb* refers to QUAO *b*, localized on atom *B.* Diagonal elements of the density matrix $\rho_{Aa,Aa}$ correspond to the population of the QUAO *Aa* and can have a maximum value of 2. The bond order (BO) between QUAO *a* on atom *A* and QUAO *b* on atom *B*, $\rho_{Aa,Bb}$, varies between 0 and 1. Kinetic bond orders (KBOs) correspond to bond orders weighted by kinetic energy integrals, and are given by Equation (2):

(2) $${KBO}_{Aa,Bb}=0.1\rho_{Aa,Bb}\left\langle Aa|-\frac{1}{2}\nabla^{2}|Bb\right\rangle$$

KBOs provide a quantitative energetic value of the covalent character of the bond formed between the QUAOs *Aa* and *Bb*. The 0.1 factor is used to compensate for the absence of the potential energy term. It is emphasized that BOs suffer from certain limitations such as negative values between bonded QUAOs due to difficulty in controlling the phases. Furthermore, certain bonding QUAO interactions may still erroneously show a negative BO, such as between the opposite carbon atoms in a benzene ring. Finally, bond orders do not provide a quantitative energy value. Therefore, only kinetic bond orders will be presented and discussed in this manuscript. It is important to emphasize that, unlike the bond orders, kinetic bond orders are consistently negative when the interaction between the QUAOs is bonding.

Overall, the QUAO method can be used to elucidate covalent bonding patterns with the following features: (1) QUAOs and their interactions are strictly derived from the ab initio molecular wavefunction (in this case HF/Jorge-TZP), without introducing any empirically defined ‘model’ wavefunction to model pre-conceived interactions. It is, therefore, a completely unbiased analysis. (2) QUAOs can be described as atomic orbitals embedded in the molecular wavefunction, which are distorted due to the tendency of the electrons to expand towards neighboring atoms (typically one but sometimes two). The resulting interactions between QUAO pairs are easier to analyze and interpret than delocalized molecular orbitals, while still being an accurate representation of the molecular wavefunction. (3) The kinetic bond orders provide a quantitative measurement of the kinetic energy lowering when atoms share electrons, which has been shown to drive covalent bonding [58].

### 2.2. Analysis of the Intermolecular Forces

The energy decomposition analysis by Su and Li [52] is used to decompose the total interaction energy ∆E between the ammonia molecule and the XC(NO_2_)_3_ compound in terms of IMF. ∆E is given as the sum of the electrostatics, exchange, repulsion, polarization, and dispersion energies (Equation (3)):

(3) $$∆E=∆E_{electrostatics}+∆E_{exchange}+∆E_{repulsion}+{∆E}_{polarization}+{∆E}_{dispersion}$$

There are several key points to emphasize regarding this method. First, unlike the KBOs described previously, the energy terms computed here are not strictly embedded in the optimized HF wavefunction of the dimer. For instance, the wavefunction of the monomers is used to compute the first three energy terms. Another key point is that only the repulsion term and polarization terms include contributions from the kinetic energy (KE), which drives covalent bonding.

### 2.3. Energy Landscape Exploration

**Figure 3:**
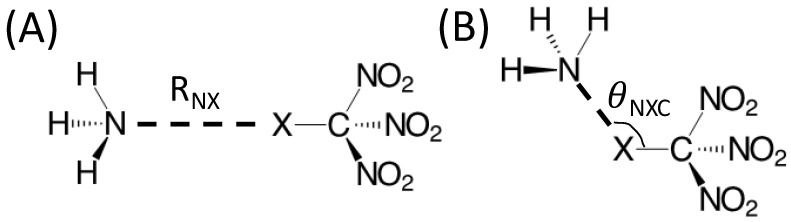
Parameters used to generate the potential energy curves of the NH_3_--XC(NO_2_)_3_ complex (X=F, Cl, Br, and I): (**A**) the N--X distance (*R_NX_*) and (**B**) the N--X-C angle ($\theta_{NXC}$).

Two sets of potential energy curves were generated for the NH_3_--XC(NO_2_)_3_ complexes, both starting from the HF-optimized structures. In the first, the N--X distance *R_NX_* is varied between 2.5 and 6.5 Å with a 0.05 Å step between 2.5 and 4.0 Å and a 0.2 Å step between 4.0 and 6.5 Å (Figure 3A). In the second, the N--X-C angle $\theta_{NXC}$ is varied from 180° to 90° with a 2° step (Figure 3B). For each point in both sets, a QUAO analysis and the evaluation of the IMFs described previously were performed. The molecular electrostatic potential maps were also generated.

## 3. Results

### 3.1. Geometry, Electrostatic Potential, and QUAO Analysis of XC(NO_2_)_3_

Bond distances and angles of the optimized XC(NO_2_)_3_ compounds are shown in Table 1. All optimized structures are local minima, as verified by Hessian calculations. The MP2-optimized C-X bond lengths are as follows: 1.303, 1.701, 1.839, and 2.074 Å for X=F, Cl, Br, and I, respectively. These distances are within 0.02 Å of the experimental X-ray diffraction values of 1.297 [41], 1.694 [36], 1.853 [41], and 2.097 [41] Å, respectively. Furthermore, these bond distances are noticeably shorter than typical C-X bonds. For instance, the C-X bond lengths in the CX_4_ compounds are 1.32 (X=F), 1.77 (X=Cl), 1.94 (X=Br) [59], and 2.16 Å (X=I) [60]. The MP2-optimized C-N bond lengths are 1.52–1.53 Å for all compounds, somewhat larger than a typical C-N single bond (~1.48 Å in amines), but consistent with experimental data [36,41]. Furthermore, the X-C-N angle and X-C-N-O dihedral angle are noticeably smaller for X=F than for the heavier halogens. They are within 0.5° and 4° of experimental values [36,41], respectively. Finally, it is noted that the two N-O bonds of the nitro groups have slightly different lengths. The N-O_1_ bond (Figure 2) is ~0.003 Å shorter than the N-O_2_ bond for the heavy halogens. The geometries observed at the HF level are generally similar to those observed at the MP2 level, with an overestimation of the C-X bond distances for the heavy halogens (up to 0.04 Å for the Br-C bond) and an underestimation of the X-C-N angles (up to ~0.9° for X=Br).

**Figure 4:**
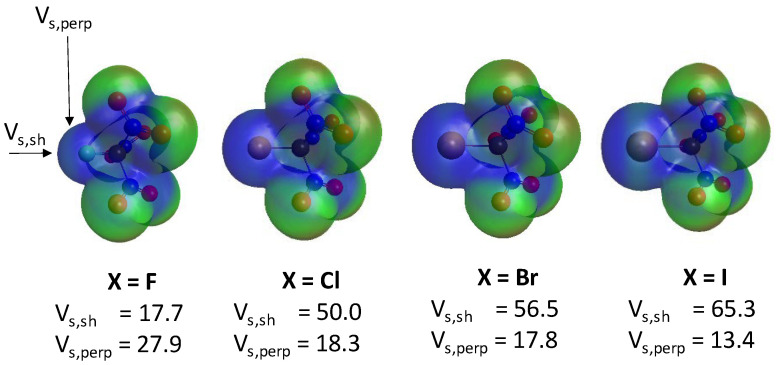
Molecular electrostatic potentials of the XC(NO_2_)_3_ compound (X=F, Cl, Br, and I). The electrostatic potential at the σ-hole (*V_s,sh_*) and above the halogen atom, perpendicularly to the X-C bond (*V_s,perp_*), are shown in kcal/mol under each contour.

The molecular electrostatic potential maps of the four optimized XC(NO_2_)_3_ compounds are shown in Figure 4. In accordance with previous observations [36], the electrostatic potential is positive on the entire surface of the halogen atoms, including fluorine. A σ-hole is only clearly visible on IC(NO_2_)_3_, where a belt with a slightly less positive potential is seen (in light green). The electrostatic potential on the surface of the halogen atom was computed at two positions: (1) along the X-C bond, where a σ-hole would be expected (labeled *V_s,sh_*), and (2) perpendicular to the X-C bond (*V_s,perp_*). It is noted that the *sh* label in *V_s,sh_* does not necessarily imply the presence of a σ-hole but rather represents the point where the electrostatic potential is computed. As expected, *V_s,sh_* increases as the halogen size increases, with a large 32 kcal/mol jump between X=F and X=Cl. On the other hand, *V_s,perp_* slightly decreases with halogen atom size, with a somewhat larger 9 kcal/mol drop between X=F and X=Cl. While *V_s,sh_* is larger than *V_s,perp_* for the heavy halogens, the reverse is observed for the fluorinated compound. Overall, the electrostatic potential is positive on the entire halogen atom surface for all halogens but still shows some anisotropy, which becomes more prominent with increasing halogen atom size. These data suggest that, while the presence of a σ-hole is not always obvious from the electron density plots, a σ-hole appears to be present for the heavy halogens, as demonstrated by the more positive *V_s,sh_* values compared to the *V_s,perp_* values. Finally, it is noted that these positive electrostatic potentials do not necessarily imply that the halogen atom has an overall positive charge, as described in the section below.

**Figure 5:**
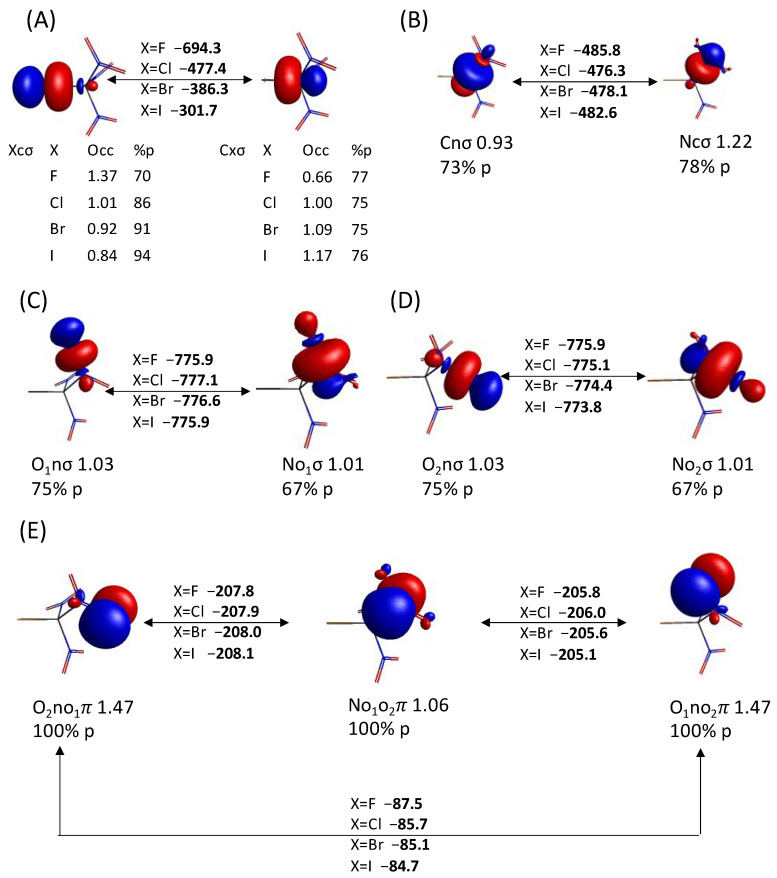
Quasi-atomic orbitals forming the σ and π bonds in XC(NO_2_)_3_ (X=F, Cl, Br, and I): (**A**) X-C σ bond, (**B**) C-N σ bond, (**C**) N-O_1_ σ bond, (**D**) N-O_2_ σ bond, and (**E**) O_1_-N-O_2_ π bond. The kinetic bond orders between QUAOs are written in bold. The QUAO labels, occupations, and % *p* character are given below each contour.

**Figure 6:**
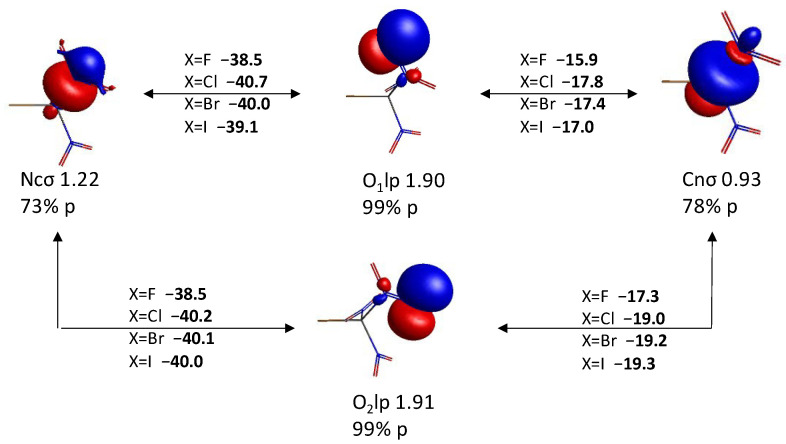
Interactions between the oxygen QUAO lone pairs (O_1_lp and O_2_lp) and the C-N σ bond.

**Figure 7:**
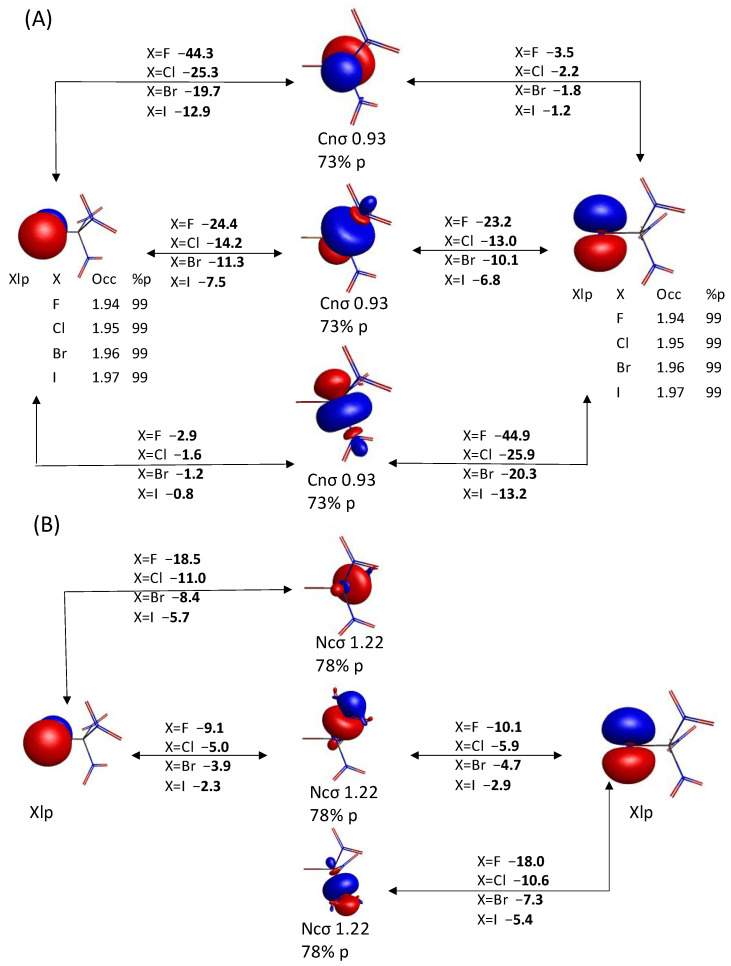
Interactions between the halogen lone pairs (Xlp) QUAOs and (**A**) the Cnσ QUAOs, and (**B**) the Ncσ QUAOs.

The symmetry unique QUAOs of the HF-optimized XC(NO_2_)_3_ compounds are shown in Figure 5, Figure 6 and Figure 7. QUAO occupations and hybridizations are shown below each contour, and the KBOs between the corresponding QUAO pairs in bold. There are 2.01–2.03 electrons *e* shared between the Xcσ QUAO and the Cxσ QUAO, forming the X-C σ bond (Figure 5A). The Cxσ QUAO has ~75% *p*-character, indicating the sp^3^ hybridization of the carbon atom for all halogen substituents X. On the other hand, the *p*-character of the Xcσ QUAO increases from 70% for X=F to 94% for X=I. These hybridization states are lower than those previously observed for the Xfσ QUAOs in the diatomic compounds XF (X=F, Cl, Br, and I) [33], where the *p*-character of the Xfσ QUAOs remains larger than 93% for all X, while the *p*-character of the Fxσ QUAO decreases from 93% to 82% as the size of X increases. These results demonstrate the persistent sp^3^ hybridization state for the bonded carbon atom in the XC(NO_2_)_3_ compounds. The occupation of the Fcσ QUAO (1.37 *e*) is larger than that of the Cfσ QUAO (0.66 *e*), indicating that the C-F σ bond is polarized towards the fluorine atom. As the size of the halogen X increases, the population of the Xcσ QUAO decreases (down to 0.84 *e* for X=I) and that of the Cxσ QUAO increases (up to 1.17 *e* for X=I), showing a reversal of the polarization of the C-X bond towards the carbon atom, in accordance with the decreasing electronegativity of the halogen atoms.

There is a total of 2.15 electrons shared between the Cnσ (0.93 *e*) and Ncσ (1.22 *e*) QUAOs, which are both sp^3^ hybridized (Figure 5B). Each C-N σ bond, therefore, has an additional 0.15 *e* compared to what would be expected for a single σ bond. As can be seen in Figure 6, the two *p* lone pairs QUAOs on the oxygen atoms of the nitro groups, O_1_lp and O_2_lp, have an occupation of 1.91 *e* and 1.90 *e*, respectively, indicating a combined loss of ~0.19 *e* for each nitro group. This value is comparable to the excess electron occupation of the C-N σ bonds, suggesting a transfer of charge from the oxygen lone pairs to the C-N bonds, mostly to the Ncσ QUAO. The N-O σ bonds share 2.04 *e*, with 1.03 *e* occupying the Onσ QUAOs and 1.01 *e* occupying the Noσ QUAOs (Figure 5C,D). These QUAOs are sp^3^ and sp^2^ hybridized, respectively. Finally, there are three π QUAOs (O_1_no_2_π, No_1_o_2_π, and O_2_no_1_π) forming the delocalized π bonds of the nitro groups (Figure 5E). The QUAOs centered on each of the oxygen atoms have 1.47 *e* while the QUAO centered on the nitrogen has 1.06 *e*, giving the expected total of four π electrons. Finally, the two *p* lone pair QUAOs on the halogen atoms (Xlp in Figure 7) also show a slight electron deficit, ranging from 0.03 *e* for X=I to 0.06 *e* for X=F. As will be discussed next, these lone pair QUAOs also display significant interactions with the C-N σ bond.

The partial atomic charges based on the QUAO occupations are reported in Table 2. The partial charge on the halogen atom is −0.25 for X=F. Therefore, while the electrostatic potential on the surface of the fluorine atom appears positive, this atom still has an overall partial negative charge. The fluorine atom receives 0.37 *e* from the carbon via the σ bond (inductive effect) but there is a combined 0.12 *e* deficit of the *p* lone pair QUAOs. The charge of the heavier halogen atom becomes increasingly positive as the size of X increases, ranging from +0.09 (X=Cl) to +0.21 (X=I). The partial positive charge of the bonded carbon atom decreases from +0.57 (X=F) to +0.08 (X=I), consistent with the polarity change of the C-X bond. The partial charges of the atoms in the nitro groups are about +0.7 for nitrogen and −0.4 for oxygen, showing only small variations when going down the halogen series (+0.02 for nitrogen and <0.01 for the oxygen atoms).

The KBO between the Xcσ QUAO and the Cxσ QUAO becomes smaller in magnitude as the size of the halogen atom X increases (from −694.3 kcal/mol for X=F to −301.7 kcal/mol for X=I), correlating with a decreasing covalent character of the bond, consistent with what was previously observed for the X-F bond in XF compounds [33]. The KBO between the QUAOs forming the C-N σ bond does not change significantly between the different halogen substituents X, with variations smaller than 2% (or 10 kcal/mol). It is noted that the KBO between the QUAOs forming the X-C σ bond is nearly 200 kcal/mol stronger than the KBO between the QUAOs forming the C-N σ bond for X=F. For X=Cl, these KBOs are nearly identical. For X=Br and I, the KBO for the C-X σ bond becomes significantly weaker than that for the C-N σ bond. The KBOs between the QUAOs forming the N-O σ and N-O π bonds areapproximately identical for all halogen atoms, showing that the covalent character of these bonds remains essentially unchanged through the halogen series.

Furthermore, Figure 6 and Figure 7 show noticeable KBOs between the QUAOs forming the C-N σ bond and the lone pairs QUAOs on the oxygen and halogen atoms, respectively. For instance, the sum of the KBOs corresponding to the O_1_lp-Cnσ and O_1_lp-Ncσ QUAO pairs reaches −54.4 kcal/mol for X=F. For the other halogenated compounds, these values are slightly larger, between −56.1 and −58.5 kcal/mol. For the O_2_lp-Cnσ and O_2_lp-Ncσ QUAO pairs, KBOs have a noticeably larger magnitude, with a value of −55.8 kcal/mol for X=F and ~−59.3 kcal/mol for the other halogens. It is interesting to note that (1) both the O_1_lp and O_2_lp QUAOs interact more strongly with the Ncσ QUAO than with the Cnσ QUAO due to a better overlap, with a difference of over 20 kcal/mol between the two; and (2) the KBOs between the O_2_lp QUAO and the Cnσ QUAOs are about 2 kcal/mol larger than those between the O_1_lp QUAOs and the Cnσ QUAOs, whereas there is essentially no difference in the interactions of these lone pairs with the Ncσ QUAOs. These KBOs and the QUAO occupations discussed previously suggest that the oxygen lone pairs do not interact with the halogen atom, but with the C-N σ bond. These interactions are energetically slightly larger for the heavy halogen atoms than for fluorine.

In addition, as demonstrated in Figure 7, there is a large KBO between the *p* lone pair QUAOs on the halogen atom Xlp and the Cnσ QUAOs. Interactions with the Ncσ QUAOs are about twice weaker. These KBOs are sensitive to the orientation of the lone pair QUAO relative to the C-N σ bond. The sum of the KBOs between the Xlp QUAOs of the halogen atom and the QUAOs forming one C-N σ bond reaches a value of ~−66 kcal/mol, −38 kcal/mol, −30 kcal/mol, and −20 kcal/mol for X=F, Cl, Br, and I, respectively.

**Figure 8:**
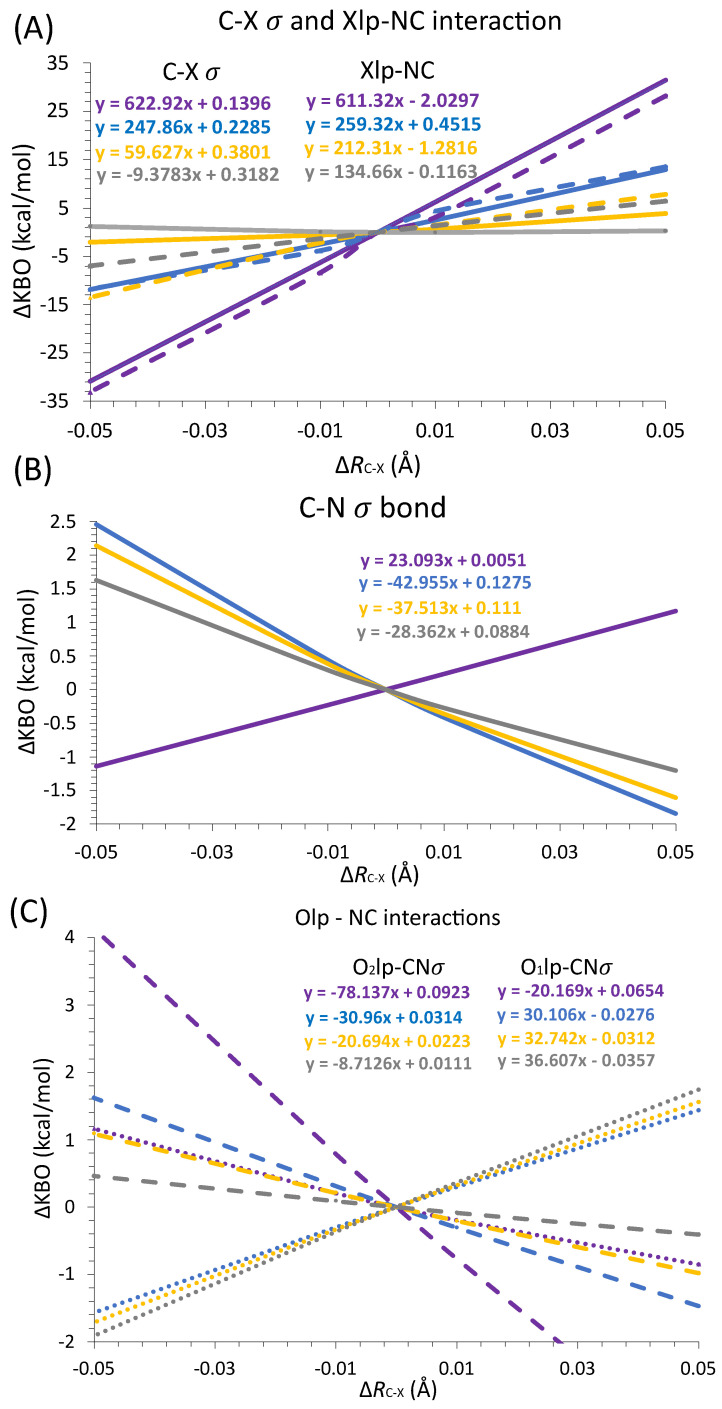
Change in KBOs as a function of Δ*R_C-X_* for (**A**) the C-X σ bonds (Cxσ-Xcσ QUAO pairs) in solid line and the Xlp interaction with the C-N σ bond (sum of the KBOs between the Xlp-Cnσ and Xlp-Ncσ QUAO pairs) in dashed line; (**B**) the C-N σ bonds (Cnσ-Ncσ QUAO pairs); and (**C**) the O_1_lp interaction with the C-N σ bond (sum of the KBOs between the O_1_lp-Cnσ and O_1_lp-Ncσ QUAO pairs) in dotted line and the O_2_lp interaction with the C-N σ bond (sum of the KBOs between the O_2_lp-Cnσ and O_2_lp-Ncσ QUAO pairs) in dashed line. The line of best fit equation is given for each data set. Color coding: Purple = F, Blue = Cl, Yellow = Br, and Gray = I.

Overall, the large KBOs and the QUAO occupations obtained show that the oxygen and halogen lone electron pairs interact strongly with the C-N σ bonds within the compound. These data offer another possible rational for the unusually short X-C distances observed in these compounds. To further investigate this, structures with varying C-X bond distances are analyzed for each XC(NO_2_)_3_ compound. The C-X bond distance of the optimized structures is elongated by 0.01 Å and 0.05 Å, corresponding to Δ*R_C-X_* values of +0.01 Å and +0.05 Å. It is also decreased by 0.01 Å and 0.05 Å, corresponding to Δ*R_C-X_* values of −0.01 Å and −0.05 Å. Δ*R_C-X_* = 0 corresponds to the equilibrium structure. The change in KBOs between the distorted geometry and the optimized geometry (ΔKBO) are computed for the different values of Δ*R*_C-X_. A negative ΔKBO value means a stronger covalent interaction compared to the equilibrium structure while a positive ΔKBO value means a weaker covalent interaction compared to the equilibrium structure. The following QUAO interactions are shown in Figure 8: the C-N σ bond (Cnσ-Ncσ QUAO pair), the C-X σ bond (Xcσ-Cxσ QUAO pair), the O_1_ lone pair with the C-N σ bonds (labeled O_1_lp-NC, and corresponding to the sum of the KBOs between the O_1_lp-Cnσ and O_1_lp-Ncσ QUAO pairs shown in Figure 6), the O_2_ lone pairs with the C-N σ bonds (labeled O_2_lp-NC, and corresponding to the sum of the KBOs between the O_2_lp-Cnσ and O_2_lp-Ncσ QUAO pairs shown in Figure 6), and the halogen lone pairs with the C-N σ bonds (labeled Xlp-NC, and corresponding to the sum of the KBOs between the Xlp-Cnσ and Xlp-Ncσ QUAO pairs shown in Figure 7). It is noted that the sum of all symmetrically equivalent bonds is considered. For instance, changes over all three C-N σ bonds of the compound are included in Figure 8B. To help quantify the changes in KBO with the *R*_C-X_ distance, the equation of the line of best fit for each data set is also given in Figure 8.

As the C-X distance decreases, the C-X σ and Xlp-NC interactions (Figure 8A) become stronger. The magnitude of the variation diminishes with halogen size, as shown by the decreasing dKBO/d*R_C-__X_* slope. For X=Br, I, the magnitude of dKBO/d*R_C-__X_* for the Xlp-NC interaction is significantly larger than for the C-X σ interaction, suggesting that the halogen lone pair interactions with the C-N σ bonds may have an increasingly larger effect on the C-X bond distance compared to the C-X σ interactions as the size of X increases. Furthermore, as the C-X distance decreases, the C-N σ bonds become slightly stabilized for X=F but destabilized for X=Cl, Br, and I (Figure 8B). Interestingly, the interaction between the O_1_ lone pair QUAOs and the C-N σ bonds (Figure 8C) become increasingly favorable with decreasing C-X bond length for the heavy halogens but are destabilized for X=F. For all halogen atoms, the O_2_lp-NC interaction (Figure 8C) is destabilized with decreasing *R_C-X_*. The dKBO/d*R_C-__X_* slopes show that the O_2_lp-NC interactions are more sensitive to halogen size than the O_1_lp-NC interactions.

In summary, it is postulated that the unusually short C-X bond length observed in the XC(NO_2_)_3_ compounds with heavy halogens may, at least in part, be explained by the increasingly favorable bonding interactions between the C-N σ bonds and the lone pairs QUAOs on the oxygen O_1_ and halogen atoms with a decreasing C-X distance. The destabilization of the O_2_lp-NC interactions with a decreasing *R_C-X_*, which is highly sensitive to the identity of the halogen atom X, partially counteracts these effects.

### 3.2. Geometry, Electrostatic Potential, and QUAO Analysis of the Dimer NH_3_--XC(NO_2_)_3_

#### 3.2.1. Geometries and Electrostatic Potentials

The MP2 and HF-optimized geometries of the NH_3_--XC(NO_2_)_3_ complexes are given in Table S1 of the Supplementary Material. It is noted that all structures are stationary points, but not all are local minima. Some dimers have a small imaginary vibrational frequency, typically corresponding to the rotation of the NH_3_ molecule along the N-X-C axis, which should have no noticeable effect on the present analysis. A brief discussion of the MP2 optimized structures is given here but similar trends are observed at the HF level. The C-X bond in the NH_3_--XC(NO_2_)_3_ dimers is about 0.005 Å shorter than in the unbound molecule for X=F, but longer for the heavier halogens (by 0.002, 0.011, and 0.052 Å for X=Cl, Br, and I, respectively). The C-N bonds undergo a slight contraction (up to 0.011 Å for X=I) upon complexation with NH_3_ for the heavy halogens. The intermolecular distance (defined as the distance *R_NX_*; Figure 3A) decreases as the size of X increases and the X-C-N angle is widened by about 0.2° for X=F, Cl, and Br but narrowed by 0.2° for X=I. The N-O bond distances are essentially the same as those reported for the XC(NO_2_)_3_ monomers.

**Figure 9:**
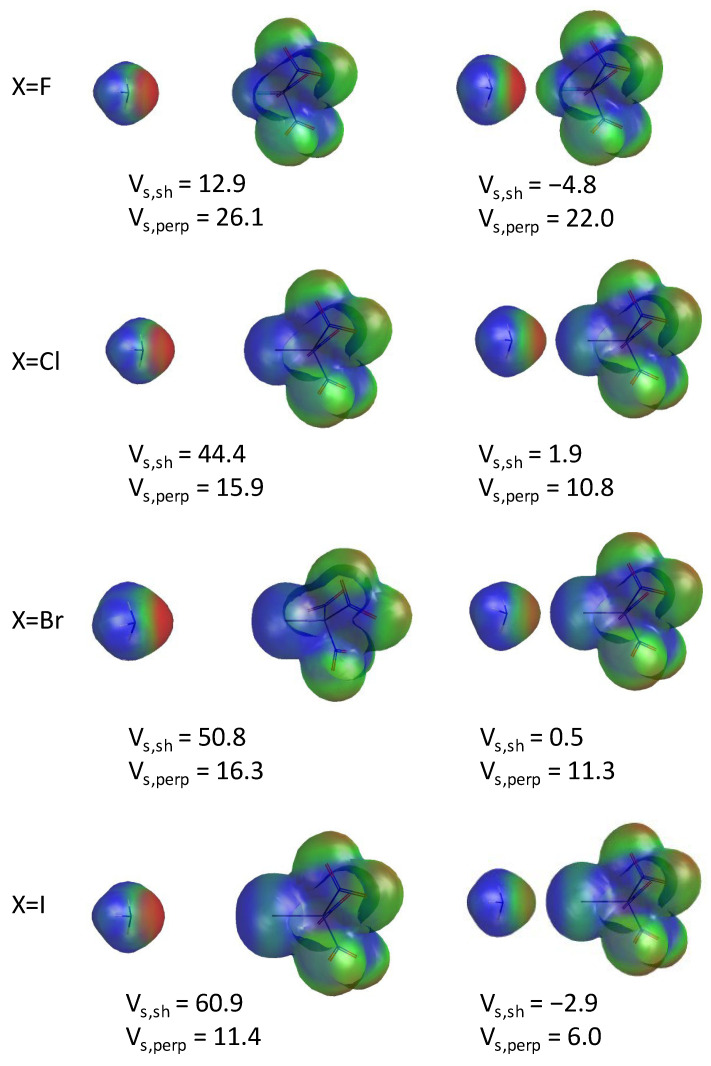
Molecular electrostatic potentials of the NH_3_--XC(NO_2_)_3_ complexes (X=F, Cl, Br, and I) at *R*_NX_=6.5 Å (left panel) and an *R*_NX_ distance that is 0.5 Å larger than the optimized distance. The electrostatic potential at the σ-hole (*V_s,sh_*) and above the halogen atom, perpendicularly to the X-C bond (*V_s,perp_*), are shown in kcal/mol under each contour.

The molecular electrostatic potential maps of the NH_3_--XC(NO_2_)_3_ dimer systems are presented in Figure 9 for two different values of the *R*_NX_ distance. A *R*_NX_ value of 6.5 Å is presented on the left panel. On the right panel, a *R*_NX_ distance that is 0.5 Å larger than the optimized distance is presented. Overall, the approach of the NH_3_ molecule induces a redistribution of the electron density in the entire system, with a large decrease in the electrostatic potential *V_s,sh_* (by 17.7 kcal/mol for X=F, and up to 63.8 kcal/mol for X=I) and, to a much smaller extent, *V_s,perp_* (by about 5 kcal/mol for all halogen atoms). For X=F, an increasing electron density on the nitrogen atom of the ammonia molecule and around the fluorine atom is observed as the intermolecular distance decreases, showing a build-up of the electron density at the N--F junction. On the other hand, for the heavier halogen atoms, there is a decrease in the electron density on the nitrogen atom, accompanied by a broadening of the belt around the halogen atom, suggesting a redistribution of the electrons, with an accumulation of charge at the belt.

#### 3.2.2. QUAO Analysis

**Figure 10:**
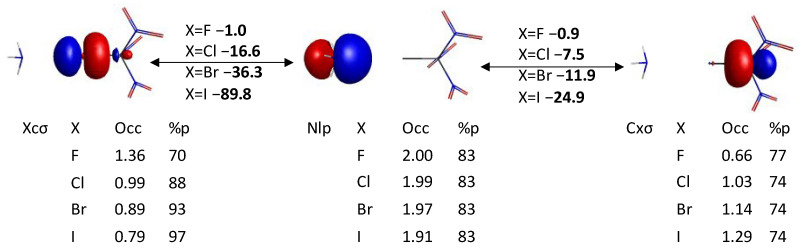
Quasi-atomic orbital analysis of the N--X-C bond. The kinetic bond orders between QUAOs are written in bold. The QUAO labels, occupations, and % *p* character are given below each contour.

In this section, the interactions between the XC(NO_2_)_3_ compound and ammonia are investigated through an analysis of the quasi-atomic orbitals. The interaction between the nitrogen lone pair (Nlp) QUAO of the ammonia molecule and the QUAOs of the C-X bond are shown in Figure 10. Similar to what was demonstrated previously for the NH_3_--XF complexes [33], there is essentially no covalent bonding between ammonia and the fluorinated compound, with no sharing of electrons between the lone pair of ammonia and the C-F bond, and corresponding KBOs in the order of −1 kcal/mol. On the other hand, the nitrogen lone pair QUAO loses from 0.01 *e* (X=Cl) to 0.09 *e* (X=I) to the XC(NO_2_)_3_ molecule, therefore demonstrating a donation of electrons from the nucleophile to the C-X bond [22,33]. It is noted that the percentage of the *p*-character of the Xcσ QUAOs (X=Cl, Br, and I) in the complex is increased by about 2% compared to the unbound molecule. Figure 10 and Table S2 of the Supplementary Material show that the occupation of the Xcσ QUAO decreases upon complex formation whereas that of the Cxσ QUAO increases. For X=F, the decreased electronic occupation of the Fcσ QUAO (−0.006 *e*) equals the increased occupation of the Cfσ QUAO (+0.006 *e*), resulting in no net change for the C-F σ bond. As we go down the halogen series, the change in population of the Cxσ QUAO becomes increasingly larger than that of the Xcσ QUAO, resulting in a larger electron population in the C-X bond compared to the unbound compound. Furthermore, the kinetic bond orders between the nitrogen lone pair of ammonia and the Xcσ and Cxσ QUAOs are significantly larger for the heavier halogens than for fluorine, steadily increasing with halogen size. In fact, the KBOs between the Nlp and Xcσ QUAOs range between −16.6 kcal/mol (X=Cl) and −89.8 kcal/mol (X=I). The KBOs between the Nlp and Cxσ QUAOs range between −7.5 kcal/mol (X=Cl) and −24.9 kcal/mol (X=I). These data potentially suggest an increasing three-center character of the halogen bond with halogen size [33]. These results are also consistent with the decreasing intermolecular distance observed as we go down the halogen series.

The KBO differences between the optimized halogen-bonded NH_3_--XC(NO_2_)_3_ complex and the optimized XC(NO_2_)_3_ molecule, ΔKBO, are shown in Table 3. Like Figure 8, the values reported correspond to the sum over all symmetrically equivalent bonds. The KBO between the Cxσ and Xcσ QUAOs becomes 3.8 kcal/mol more negative upon complexation with ammonia for X=F, indicating an increased stabilization of the C-F bond due to electron sharing. On the other hand, for X=Cl, Br, and I, these KBOs become less negative (increasing by +3.6, +4.8, and +6.9 kcal/mol, respectively), overall consistent with the observed elongation of the C-X bonds.

While the QUAOs forming the X-C σ bond undergo the most significant changes in both KBOs and occupations, the C-N σ bonds are also noticeably affected. As shown in Table S2 of the Supplementary Material, the population of the C-N σ bonds (summed over all three C-N bonds) slightly vary by +0.001 *e*, −0.003 *e*, −0.010 *e,* and −0.032 *e*, for X=F, Cl, Br, and I, respectively. The corresponding ΔKBO values are +1.8 kcal/mol, +1.0 kcal/mol, −2.1 kcal/mol, and −13.2 kcal/mol, respectively, which indicates a slight decrease in the covalent character of the bonds upon complex formation for X=F and Cl and a slight increase for X=Br and I.

The ΔKBO values of the O-N σ bonds and the O-N-O π systems all increase slightly upon complex formation. Notably, the QUAO interactions involving the O_1_ atoms appear more affected by halogen size than those involving the O_2_ atoms. For instance, while the ΔKBO value for the O_2_nσ-No_2_σ QUAO pairs is not significantly affected by the identity of the halogen atom, ranging from +1.4 kcal/mol to +2.4 kcal/mol, the ΔKBO value for the O_1_nσ-No_1_σ QUAO pairs is slightly negative for X=F (−0.9 kcal/mol) and becomes more positive as the size of X increases (up to +5.6 kcal/mol for X=I). Similarly, the ΔKBO value between the O_1_lp QUAOs and the QUAOs forming the C-N σ bonds (the Cnσ and Ncσ QUAOs) increases by ~12 kcal/mol from X=F to X=I, compared to a ~5 kcal/mol increase for the O_2_lp QUAO. These results show a weakening of the interaction between the oxygen lone pair QUAOs and the C-N σ bonds.

As shown by the negative ΔKBO values, the interactions between the halogen lone pairs Xlp and the C-N σ bond are strengthened upon complexation with ammonia for X=F, Cl, and Br. The ΔKBO values range from −6.1 kcal/mol (X=F) to −3.3 kcal/mol (X=Br). Interestingly, for X=I, these interactions are overall weakened (by 6.5 kcal/mol). When considering both the halogen and oxygen lone pair interactions with the C-N σ bond, these data show that, for X=F, the stabilizing Xlp interaction with the C-N σ bond is larger than the destabilizing interaction between the Olp QUAOs and the C-N σ bond. For the larger halogens, the reverse is true. Finally, it is noted that the changes in KBOs and QUAO populations are significantly larger for iodine than for the other halogens. As shown in Table S3 of the Supplementary Material, the large structural distortions of the IC(NO_2_)_3_ molecule upon complexation with ammonia strongly affect the electronic properties of the complex, partly accounting for the unusual behavior of iodine.

In summary, the formation of a N--X bond with a covalent character is observed for X=Cl, Br, and I, but not for X=F. The degree of covalency of the N--X-C interaction, corresponding to the kinetic energy lowering due to electron sharing between the atoms (and quantified using the KBOs), increases from Cl to I. The electron donation from the lone pair of the ammonia molecule for X=Cl, Br, and I leads to a matching increase in the electronic population of the C-X bond. Interestingly, the adjacent C-N σ bonds undergo small but noticeable changes in terms of occupations and KBOs. Finally, noticeable changes in the interactions of the oxygen and halogen lone pairs with the QUAOs involved in the C-N σ bond are observed, showing a significant contribution to the electronic properties of the system.

#### 3.2.3. Intermolecular Forces

Table 4 shows a decomposition of the interaction energy between the NH_3_ and XC(NO_2_)_3_ molecules for the four optimized dimers in terms of intermolecular forces (IMFs), as described in the methods section. The HF-optimized structures were used to be consistent with the QUAO analysis, but the trends discussed in this section are also seen at the MP2-optimized geometry (Table S4 of the Supplementary Material). According to Table 4, the magnitude of all five IMFs becomes larger as the size of the halogen atom increases. While the polarization and dispersion interactions tend to keep a smaller magnitude (mostly in the order of 1–2 kcal/mol), the electrostatic, exchange, and repulsion interactions become increasingly dominant with increasing halogen size. The complex interplay between the different IMF and their role in the halogen bond formation (notably, the importance of exchange–repulsion and charge transfer) has been discussed in several studies [25,26,61,62]. In this work, the contribution of the kinetic energy (KE) in halogen bond formation is highlighted.

**Figure 11:**
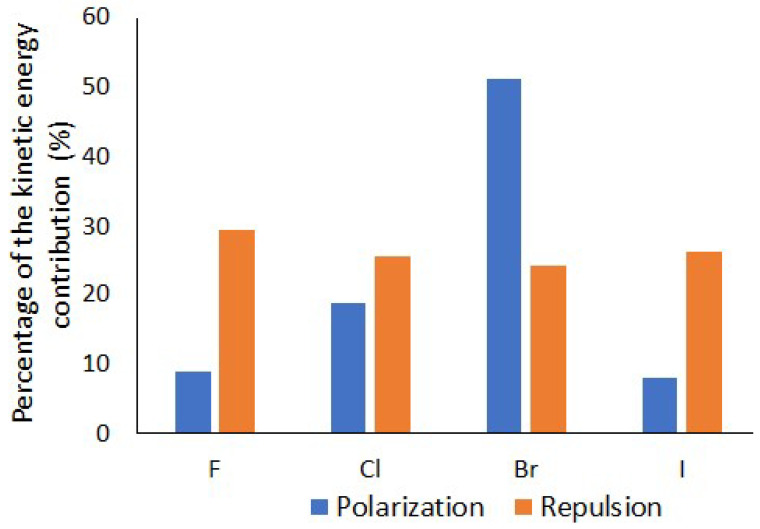
Percentage of the kinetic energy contribution to the polarization and repulsion energy terms from the IMF analysis of the NH_3_--XC(NO_2_)_3_ complex.

As described in the previous section, there is a noticeable amount of charge transfer and a significant kinetic bond order between the nitrogen lone pair QUAO of the ammonia molecule and the QUAOs forming the C-X σ bond, showing an increasing covalent character of the N--X-C bond with the increasing size of X. The kinetic bond orders (Equation (2)) represent a quantitative measure of the covalent character of a bond, which is driven by kinetic energy (KE) lowering upon electron sharing. The KE contribution to the different IMFs is presented here. As highlighted in the method section, only the polarization and repulsion energies have a KE contribution. As shown in Tables S5–S8 of the Supplementary Material, this contribution is positive for the repulsion term (increases the total energy) and negative for polarization (decreases the total energy), both becoming larger with halogen size. On the other hand, the sum of the other terms (electron exchange, Coulomb, nuclear repulsion, and electron–nucleus potential energies), contributing to both the repulsion and polarization energy, is smaller, with an opposite sign. Figure 11 shows the percentage of the magnitude of the kinetic energy term to the sum of the magnitudes of all terms (kinetic energy, electron exchange, Coulomb, nuclear repulsion, and electron–nucleus potential energies) in the polarization and repulsion energies. The % contribution of the kinetic energy remains nearly constant for the repulsion term but shows a steep increase for the polarization term, from about 9% for X=F to over 50% for X=Br. Surprisingly, it goes back down to about 8% for X=I. This last result could potentially be due to the use of an effective core potential for iodine. As will be discussed in more detail in the next section, the polarization energy term comprises the covalent bonding character of the σ-hole interaction.

### 3.3. NH_3_--XC(NO_2_)_3_ Energy Landscape Exploration

#### 3.3.1. Intermolecular Distance

**Figure 12:**
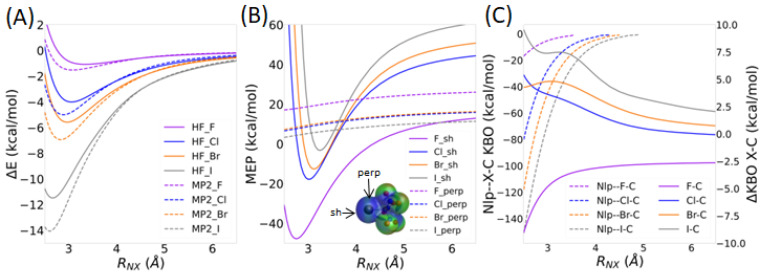
Properties of the NH_3_--XC(NO_2_)_3_ complexes as a function of the *R*_NX_ distance (Å). (**A**) MP2 and HF interaction energy (kcal/mol). (**B**) Molecular electrostatic potential (kcal/mol) at the σ-hole (*V_sh_*) of the halogen atom and above the halogen atom, perpendicular to the X-C bond (*V_perp_*) as shown in inset. (**C**) Dashed curves (left y-axis): Sum of the KBOs between Nlp-Xcσ and Nlp-Cxσ QUAO pairs. Solid curves (right y-axis): Difference in KBO for the Cx-Xcσ QUAO pair between the NH_3_--XC(NO_2_)_3_ complex and the XC(NO_2_)_3_ monomer.

The potential energy landscape of the NH_3_--XC(NO_2_)_3_ complex was investigated by computing the HF and MP2 interaction energies, molecular electrostatic potentials, and KBOs at *R*_NX_ distances varying between 2.5 and 6.5 Å, with a 0.05 Å step, while maintaining the N--X-C angle at 180° (Figure 3A). The MP2 potential energy curves in Figure 12A show a local minimum for all complexes at intermolecular distances of ~2.60 Å (X=I), ~2.85 Å (X=Br), ~2.90 Å (X=Cl), and ~3.10 Å (X=F). At the HF level, these distances are overestimated, ranging between ~2.65 Å (X=I) and ~3.35 Å (X=F). As we go down the halogen series, the interaction energy at the minimum increases from −1.5 kcal/mol for X=F, to −14.1 kcal/mol for X=I. The HF level of theory underestimates the interaction energy by ~0.4 kcal/mol (X=F) to ~2.5 kcal/mol (X=I).

As the *R_NX_* distance is shortened, *V*_s,sh_ decreases significantly whereas *V*_s,perp_ remains approximately constant (Figure 12B). The former changes from positive to negative at *R_NX_* distances of ~4.5 Å (X=F), ~3.55 Å (X=Cl), ~3.50 Å (X=Br), and ~3.40 Å (X=I). Furthermore, *V*_s,sh_ shows a minimum at *R_NX_* distances of ~2.75 Å, ~3.00 Å, ~3.10 Å, and ~3.25 Å for X=F, Cl, Br, and I, respectively. For X=F, *V*_s,sh_ is smaller than *V*_s,perp_ for all *R_NX_* distances. More importantly, the change in *V_s,sh_* with decreasing *R_NX_* distance is nearly the same for all halogen atoms, with a drop of ~65–69 kcal/mol between the *V_s,sh_* value of the monomer and the minimum of the electrostatic potential curve shown in Figure 12B. The most significant difference between X=F and the heavier halogens is that the *V_sh_* value of the FC(NO_2_)_3_ monomer is significantly lower than that of the other halogenated systems (Figure 4). Therefore, the *V_sh_* curve is shifted to lower values.

The KBOs between the nitrogen lone pair QUAO Nlp and the X-C bond, defined as the sum of the KBOs between the Nlp-Xcσ and Nlp-Cxσ QUAO pairs, are presented in Figure 12C in a dashed line, with the scale shown on the left vertical axis. The covalent interaction between the Nlp QUAO and the QUAOs forming the X-C bond becomes larger with decreasing intermolecular distance. This effect is enhanced as we go down the halogen series, as explained previously. Interestingly, the covalent character of the X-C bond, shown with the solid curve in Figure 12C, increases as the R_NX_ distance decreases for X=F (ΔKBO becomes more negative), but decreases for the heavier halogens (ΔKBO becomes more positive). These changes become more noticeable at short *R*_NX_ distances for X=F (*R_NX_* ~3.5 Å) and at larger *R*_NX_ distances (*R_NX_* ~4.5 Å) for X=Cl, Br, and I.

**Figure 13:**
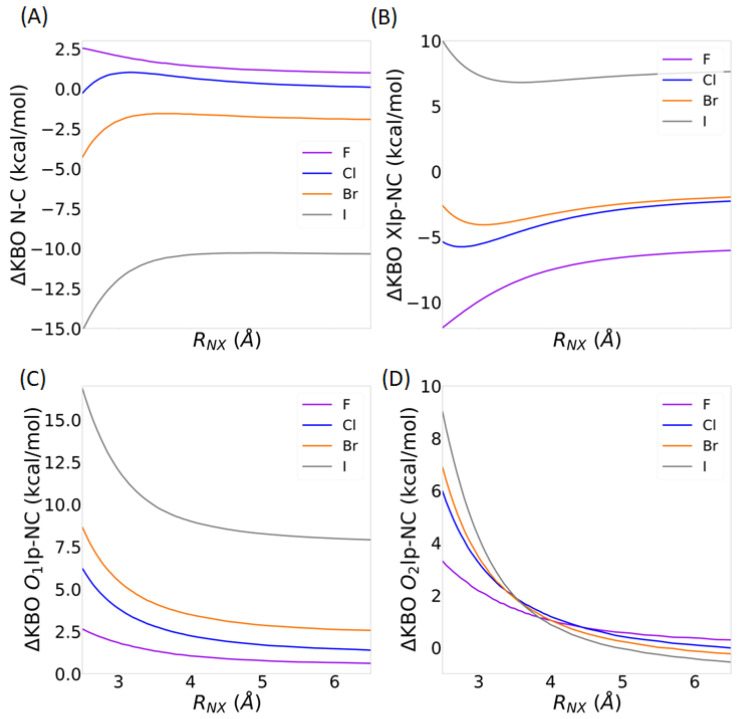
Difference in KBOs between the NH_3_--XC(NO_2_)_3_ and XC(NO_2_)_3_ systems as a function of the *R*_NX_ distance for (**A**) the C-N σ bond, corresponding to the Cnσ and Ncσ QUAO pairs; (**B**) the halogen lone pair and C-N σ bond, corresponding to the sum over all Xlp-Cnσ and Xlp-Ncσ QUAO pairs; (**C**) the oxygen O_1_ lone pair and the C-N σ bond, corresponding to the sum over all O_1_lp-Cnσ and O_1_lp-Ncσ QUAO pairs; and (**D**) the oxygen O_2_ lone pair and C-N σ bond, corresponding to the sum over all O_2_lp-Cnσ and O_2_lp-Ncσ QUAO pairs.

Figure 13 shows the change in KBOs between the dimer and optimized propeller monomer for the QUAO pairs corresponding to the C-N σ bond and those corresponding to the oxygen and halogen lone pair QUAO interactions with the C-N σ bond, as a function of the *R_NX_* distance. Note that the sum of all symmetrically equivalent bonds is again considered (similar to Figure 8 and Table 3). The results show the widespread effect of NH_3_ binding on the bonding patterns of the XC(NO_2_)_3_ compounds. The kinetic bond order of the C-N σ bonds (Figure 13A) does not vary significantly until *R*_NX_ becomes smaller than about 3 Å. The kinetic bond order for the C-N σ bond is lowered with decreasing *R_NX_* distances for X=Cl, Br, and I and increased for X=F. These results, therefore, show a slight kinetic energy destabilization of the C-N bonds at short *R_NX_* distances for X=F and a stabilization of these bonds for the heavier halogens. The KBOs between the halogen lone pairs Xlp and the C-N σ bonds (Figure 13B) are also somewhat sensitive to the intermolecular distance, with ΔKBO values slowly decreasing as *R_NX_* decreases. Minima at *R_NCl_* = 2.8 Å, *R_NBr_* = 3.1 Å, and *R_NI_* = 3.6 Å are seen for the heavier halogens. For X=F, no minimum is observed. The covalent interaction between the halogen lone pairs QUAOs and the C-N σ bonds are, therefore, enhanced as *R*_NX_ decreases.

Figure 13C,D display the change in KBOs corresponding to the O_1_lp and O_2_lp interactions with the QUAOs forming the C-N σ bonds, respectively. These KBOs show a steep increase as the intermolecular distance decreases. Between *R_NX_* = 4.0 Å and *R_NX_* = 2.5 Å, the ΔKBO values for the O_1_lp-NC interactions increase by 1.6, 4.0, 5.1, and 7.8 kcal/mol for X=F, Cl, Br, and I, respectively. The ΔKBO values for the O_2_lp-NC interactions increase by slightly larger values of 2.3, 4.8, 5.8, and 8.1 kcal/mol, respectively. The covalent interaction between the oxygen lone pairs QUAOs and the C-N σ bonds, therefore, become rapidly destabilized with a decreasing *R*_NX_ distance.

**Figure 14:**
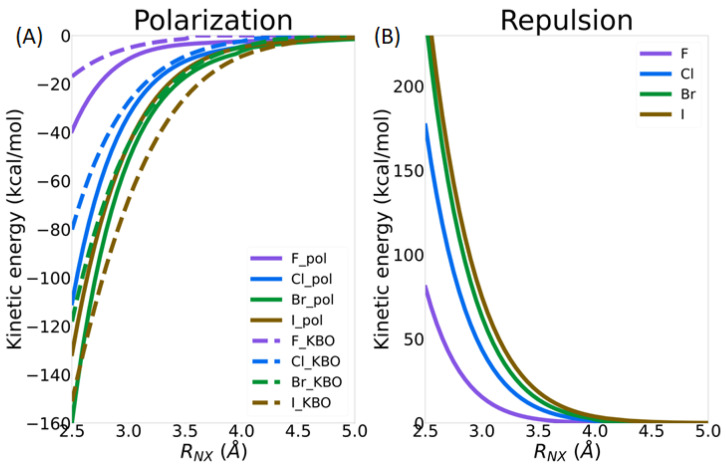
Contribution of the kinetic energy in NH_3_--XC(NO_2_)_3_ (X=F, Cl Br, and I) as a function of the *R*_NX_ distance: (**A**) kinetic energy contribution to the polarization term in the IMF analysis (solid line) and sum of the kinetic bond orders between the QUAO pairs Nlp-Xcσ and Nlp-Cxσ (dashed line); and (**B**) kinetic energy contribution to the repulsion term in the IMF analysis.

The decomposition of the total MP2 energy in terms of intermolecular forces as a function of the *R_N_*_X_ distance (Figures S2 and S3 in the Supplementary Materials) again shows the overall dominance of the electrostatic, exchange, and repulsion terms in terms of magnitude. There is, again, a complex interplay between the different intermolecular forces, and they must all be considered in order to obtain an accurate picture of the σ-hole interaction. In this section, the role of the kinetic energy (contributing to the polarization and repulsion terms) is again highlighted. Figure 14A shows that the sum of the KBOs between the Nlp-Xcσ and Nlp-Cxσ QUAO pairs and the kinetic energy contribution to the polarization term in the IMF analysis follow each other closely, becoming more negative as the intermolecular distance decreases. The covalent character of the N--X-C bond, driven by the lowering of the kinetic energy between the nitrogen lone pair QUAOs and the QUAOs forming the C-X σ bond, therefore appears to be included in the small polarization term in the IMF decomposition scheme used here. It is noted that the importance of polarization in describing σ-hole interactions was previously established [5,24]. Figure 14B shows the kinetic energy component of the repulsion term, which becomes larger with decreasing distance. Therefore, the lowering of the kinetic energy contribution to the polarization energy is counterbalanced by an increase in the kinetic energy contribution to the repulsion energy term.

In summary, the halogen bond formation induces widespread changes in the covalent bonding interactions of the XC(NO_2_)_3_ system. In addition to the large stabilization of the N--X-C bond, a significant kinetic energy destabilization of the bonding interactions between the oxygen lone pairs and the C-N σ bonds occurs, accompanied by a smaller stabilization of the interactions between the halogen lone pairs and the C-N σ bonds. Furthermore, the covalent character of the N--X-C bond, driven by the kinetic energy lowering of the interaction between the nitrogen lone pair QUAOs and the QUAOs forming C-X σ bond, appears to be embedded in the polarization energy term in the IMF analysis used here. It is, however, emphasized that the polarization term has a much smaller magnitude than the dominating electrostatics and exchange terms.

#### 3.3.2. Directionality of the σ-Hole Interaction

**Figure 15:**
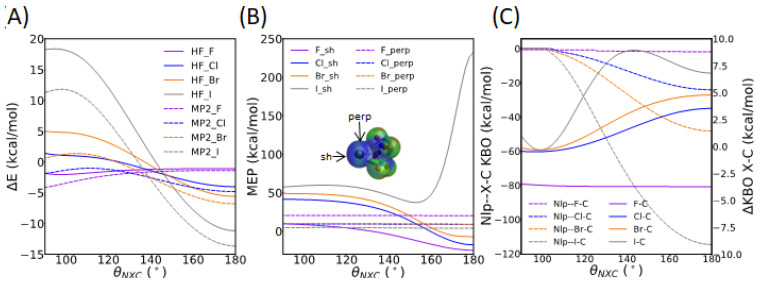
Properties of the NH_3_--XC(NO_2_)_3_ complex as a function of the *θ*_NXC_ angle. (**A**) MP2 and HF interaction energy (kcal/mol). (**B**) Molecular electrostatic potential (kcal/mol) at the σ-hole (*V_sh_*) of the halogen atom and above the halogen atom, perpendicular to the X-C bond (*V_perp_*), as shown in inset. (**C**) Dashed curves (left y-axis): Sum of the KBOs between Nlp-Xcσ and Nlp-Cxσ QUAO pairs. Solid curves (right y-axis): Difference in KBO for the Cxσ-Xcσ QUAO pair between the NH_3_--XC(NO_2_)_3_ complex and the XC(NO_2_)_3_ monomer.

The potential energy landscape of the NH_3_--XC(NO_2_)_3_ complex was further investigated by computing the HF and MP2 interaction energies, molecular electrostatic potentials, and KBOs at N--X-C angles *θ*_NXC_ varying between 90 and 180°, with a 2° step, while maintaining the internal geometries and *R*_NX_ distances identical to those of the optimized dimers (Figure 3B). Figure 15A shows that the total interaction energy Δ*E* is the lowest at the 180° angle for the three heavy halogens, as expected for the σ-hole interaction. Δ*E* overall increases steeply as the angle is reduced. The energy difference between the bent and linear configurations becomes larger as we go down the halogen series. For X=F, Δ*E* slightly decreases (by about 2 kcal/mol) as the angle *θ*_NXC_ varies between 180° and 90°. The electrostatic potential maps of the NH_3_--XC(NO_2_)_3_ systems at *θ*_NXC_ = 180° and at *θ*_NXC_ = 136° (Figure S4 of the Supplementary Material) demonstrate that, for the heavy halogens, the electron density on the nitrogen atom of NH_3_ is larger at the bent *θ*_NXC_ angle than at the linear *θ*_NXC_ angle, emphasizing the importance of a linear configuration for a σ-hole interaction. Figure 15B shows an increase in *V_s,sh_* by 34, 59, and 56 kcal/mol as *θ*_NXC_ is reduced from 180° to 90° for X=F, Cl, and Br, respectively. Note that, for X=I, *V*_s,sh_ decreases steadily by about 20 kcal/mol between *θ*_NXC_ = 90° and *θ*_NXC_ = 154° and abruptly increases by 195 kcal/mol between 154° and 180°. On the other hand, the value of *V*_s,perp_ remains nearly constant for all halogen atoms. For X=Cl and X=Br, *V_s,sh_* is larger than *V_s,perp_* at *θ*_NXC_ angles smaller than 158° and 154°, respectively. For X=F, *V_s,sh_* is smaller than *V_s,perp_* at all geometries, whereas the reverse is true for X=I. Overall, the changes in the molecular electrostatic potential upon bending are similar for all halogens, except for the large maximum obtained for X=I at the linear geometry.

Figure 15C shows the sum of the KBOs between the nitrogen lone pair QUAOs and the QUAOs forming the C-X bond, as well as the change in KBO between the QUAOs forming the C-X bond, as a function of *θ*_NXC_. The sum of the KBOs between the Nlp-Xcσ and Nlp-Cxσ QUAO pairs clearly shows that the covalent character of the N--X-C bond is the highest at *θ*_NXC_ = 180° and steeply decreases as *θ*_NXC_ decreases for the heavy halogens. Furthermore, the KBOs between the corresponding Xcσ and Cxσ QUAOs overall become less positive as *θ*_NXC_ is reduced from 180° to 90°, showing that the C-X bond is weakened in the linear configuration. Interestingly, for X=I, the ΔKBO value for the C-X bond reaches a maximum at *θ*_NXC_ = 144°. For X=F, the KBO between the nitrogen lone pair and the C-F bond remains at about 0, showing no covalent interaction at any orientation. Furthermore, the KBO between the Fcσ and Cfσ QUAOs is not affected by the *θ*_NXC_ angle.

**Figure 16:**
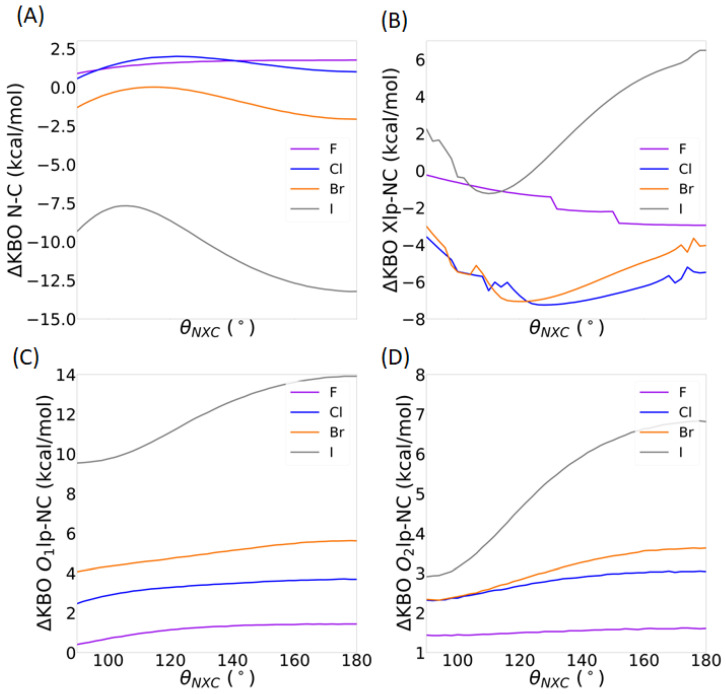
Difference in KBOs between the NH_3_--XC(NO_2_)_3_ and XC(NO_2_)_3_ systems as a function of *θ*_NXC_. (**A**) The C-N σ bond, corresponding to the Cnσ and Ncσ QUAO pairs. (**B**) The halogen lone pair and C-N σ bond, corresponding to the sum over all Xlp-Cnσ and Xlp-Ncσ QUAO pairs. (**C**) The oxygen O_1_ lone pair and C-N σ bond, corresponding to the sum over all O_1_lp-Cnσ and O_1_lp-Ncσ QUAO pairs. (**D**) The oxygen O_2_ lone pair and C-N σ bond, corresponding to the sum over all O_2_lp-Cnσ and O_2_lp-Ncσ QUAO pairs corresponding to the sum over all O_2_lp-Cnσ and O_2_lp-Ncσ QUAO pairs.

Figure 16 shows the change in KBOs between the dimer and optimized propeller monomer, as a function of the *θ*_NXC_ angle. Note that the sum of all symmetrically equivalent bonds is again presented, similar to the previous sections. The ΔKBOs between the QUAOs forming the C-N σ bonds are shown in Figure 16A. As discussed in Section 2.2 of the results, ΔKBOs are slightly positive (up to 2 kcal/mol) for X=F and Cl, showing a slight lowering of the covalent character of the C-N σ bond. They are, however, negative for X=Br and I, showing a strengthening of the covalent character of the bond. Overall, ΔKBO values for the C-N σ bond tend to be the lowest at the linear geometry for the heavy halogens, and at the bent geometry for X=F. Although variations between the different angles are quite mild for X=F, Cl, and Br (~1 kcal/mol), they are noticeably larger for X=I (about 4 kcal/mol). As explained previously, the large values obtained for X=I may, in part, be explained by the larger geometrical distortion of the propeller molecule when it is bound to NH_3_ (Table S3 of the Supplementary Material).

On the other hand, the halogen lone pair QUAO interactions with the C-N σ bond (Figure 16B) are quite sensitive to *θ*_NXC_. For X=F, the ΔKBO value increases steadily from −2.95 to −0.24 kcal/mol upon the lowering of *θ*_NXC_, showing a weakening of the halogen lone pair interactions with the C-N σ bonds. For X=Cl, Br, and I, a minimum is observed at *θ*_NXC_ values of 128°, 122°, and 110°, respectively. ΔKBO values between *θ*_NXC_ = 180° and these minima are lowered by 1.8 kcal/mol for X=Cl, 3.0 kcal/mol for X=Br, and 7.7 kcal/mol for X=I, indicating a strengthening of these interactions upon bending. It is noted that some small discontinuities in these curves are observed, which are possibly explained by the fact that KBOs with very small magnitudes do not get printed in the GAMESS output file. Some of the individual KBOs may fall under the printout threshold at certain angle values and, therefore, are not considered in the overall ΔKBO values shown here. The ΔKBOs between the oxygen lone pair QUAOs and the QUAOs forming the C-N σ bond (Figure 16C,D) are positive at all values of *θ*_NXC_, showing a destabilization of these interactions upon complexation with NH_3_, as discussed in Section 2.2 of the results. This destabilization is slightly enhanced in the linear geometry compared to the bent geometry for X=F, Cl, and Br (within ~1 kcal/mol). For X=I, there is a 4 kcal/mol difference in ΔKBO between *θ*_NXC_ = 180° and *θ*_NXC_ = 90°.

**Figure 17:**
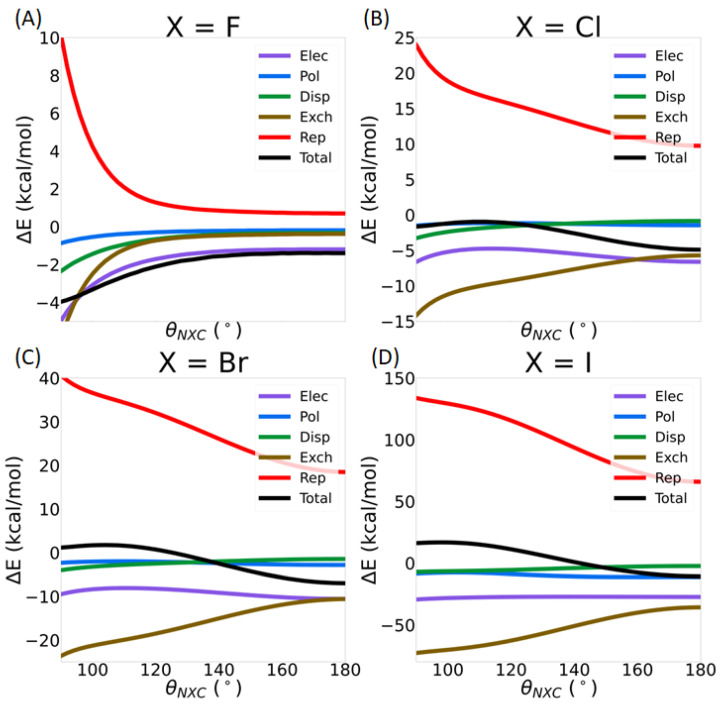
Decomposition of the total interaction energy between the NH_3_ molecule and the XC(NO_2_)_3_ compound as a function of *θ*_NXC_: (**A**) X=F, (**B**) X=Cl, (**C**) X=Br, and (**D**) X=I.

**Figure 18:**
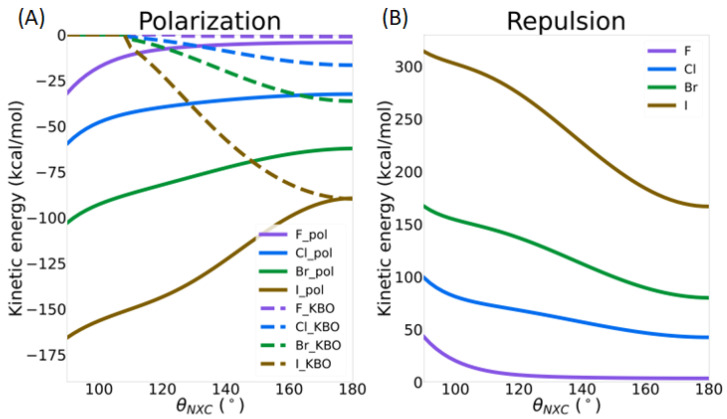
Contribution of the kinetic energy in NH_3_--XC(NO_2_)_3_ (X=F, Cl, Br, and I) as a function of the *θ*_NXC_ angle: (**A**) kinetic energy contribution to the polarization term in the IMF analysis (solid line) and sum of the kinetic bond orders between the QUAO pairs Nlp-Xcσ and Nlp-Cxσ (dashed line); and (**B**) kinetic energy contribution to the repulsion term in the IMF analysis.

Figure 17 shows the intermolecular interaction energies as a function of the *θ*_NX*C*_ angle. A detailed analysis of these IMFs is beyond the scope of this paper, but one can see that electrostatics and exchange have the largest magnitude. Furthermore, the magnitude of all IMFs increases with halogen size. The electrostatic, polarization, and dispersion energy terms do not change significantly with the *θ*_NXC_ angle. On the other hand, the exchange and repulsion terms show the largest increase in magnitude upon bending. Finally, a striking difference in the behaviors of these two energy components between X=F and the heavy halogens as *θ*_NX*C*_ is reduced can be observed: For X=F, the IMF are nearly constant until *θ*_NX*C*_ reaches about 110°. On the other hand, for the heavier halogens, slight deviations in the *θ*_NX*C*_ angle from 180° generate noticeable changes in the repulsion and exchange energy terms. The contribution of the kinetic energy is, once again, highlighted in this discussion. Figure 18 shows the contribution of the kinetic energy to the polarization (Figure 18A) and repulsion terms (Figure 18B) as a function of the *θ*_NXC_ angle. KBOs between the nitrogen lone pair QUAOs and the QUAOs forming the C-X σ bond are also shown in Figure 18A. The magnitude of the KE contribution to the polarization energy increases as the *θ*_NXC_ angle decreases, with the changes becoming larger with the halogen atom size. On the other hand, the KBOs between the Nlp QUAOs and the QUAOs forming the C-X bond (X=Cl, Br, and I) decrease as *θ*_NXC_ decreases, becoming essentially 0 at an angle of about 110°, as discussed previously (Figure 15C). These two contributions are similar at linear angles but drift apart as the angle decreases. Therefore, it is hypothesized that, at the linear *θ*_NXC_ angle, the formation of the covalent N--X-C bond dominates the KE contribution to the polarization term, but, as *θ*_NXC_ decreases, this contribution diminishes. Both Figure 14B and Figure 18B show that the KE contribution to the repulsion term follows the same pattern as the overall repulsion energy, suggesting that KE may be a dominant factor in this term.

In summary, the directionality of the σ-hole interaction in the NH_3_-XC(NO_2_)_3_ complexes (X=Cl, Br, and I) is reflected in the lowering of the total interaction energy and in the lowering of the electrostatic potential at the σ-hole position as *θ*_NXC_ increases. The increasing covalent character of the N--X-C bond is also revealed in the increasing KBOs between the nitrogen lone pair QUAO of NH_3_ and the QUAOs forming the C-X bond as *θ*_NXC_ becomes more linear. Overall, both the oxygen lone pair QUAO interactions with the C-N σ bond tend to be more destabilized in the linear geometry compared to the bent structures. The halogen lone pairs interactions with the C-N σ bond tend to be energetically stabilized in the bent structure. Finally, the present analysis suggests that the kinetic energy can provide valuable insight into the formation of halogen bonds.

## 4. Computational Methods

All calculations were performed using the GAMESS software version 2024.R1 [63,64,65]. The XC(NO_2_)_3_ molecules (X=F, Cl, Br, and I) and NH_3_--XC(NO_2_)_3_ complexes were optimized with C_3_ symmetry at the MP2 level of theory and at the Hartree–Fock (HF) level of theory using the Jorge–TZP basis set [66,67] in conjunction with a Douglas–Kroll Hamiltonian [68]. The atomic basis sets were downloaded from the basis set exchange website [69,70,71]. The HF QUAOs and molecular electrostatic potential maps of the HF-optimized structures were plotted using the MacMolPlot software [72]. A contour value of 0.007 electrons/bohr^−3^ with a maximum potential value mapped of 0.05. a.u were used. Electrostatic potential values on the halogen surface were computed at a distance corresponding to their van der Waals radius from the nucleus (*R*_vdw_ = 1.47, 1.75, 1.85, and 1.98 Å for X=F, Cl, Br, and I, respectively). For the analysis of the IMFs, the MP2/Def2-TZVP [73] level of theory with an effective core potential for Iodine [74] was used, as the use of a relativistic Douglas–Kroll Hamiltonian is not supported.

## 5. Conclusions

In conclusion, the electronic structure of the halogenated compounds XC(NO_2_)_3_ (X=F, Cl, Br, and I) was investigated. These systems are unique because of their unusually short C-X distance (which is not observed for similar carbon compounds with strong electron withdrawing groups) and because the entire halogen atom surface appears to be positive. Although not obvious at first glance, the analysis of the electrostatic potential of these compounds does show the presence of a σ-hole for the heavy halogens. The quasi-atomic orbital analysis, which provides insight into the covalent character of the bonding interactions, shows that the fluorine atom is negatively charged despite the positive electrostatic potential around the surface, while the heavier halogens are positively charged. Most importantly, this analysis shows no bonding interaction between the oxygen of the nitro group and the halogen atoms. However, the oxygen lone pair and halogen lone pair QUAOs strongly interact with the C-N σ bonds, as shown by the kinetic bond orders and QUAO occupations. This work suggests that the unusually short C-X bond of these compounds may be due (at least in part) to covalent interactions between the oxygen/halogen lone pair QUAOs and the C-N σ bonds.

Furthermore, the halogen-bonded NH_3_--XC(NO_2_)_3_ complexes were investigated at their optimized geometry and at varying *R*_NX_ distances and *θ*_NXC_ angles in terms of QUAOs and IMF. The quasi-atomic orbital analysis shows the increasing covalent character of the N--X-C bond as the angle becomes linear, which is captured in the polarization energy term of the IMF. It also shows that the interactions between the oxygen lone pair QUAOs and the C-N σ bond tend to be destabilized while those between the halogen lone pair QUAOs and the C-N σ bond (except for X=I) tend to be stabilized. The analysis of the kinetic bond orders and intermolecular forces suggests that the covalent character of the N--X-C interaction is captured in the polarization energy term. The QUAO analysis provides a novel tool in characterizing halogen bonding, beyond electrostatic potentials.

## Figures and Tables

**Table 1:** Bond distances (Å), angles (°) of the XC(NO_2_)_3_ (X=F, Cl, Br, and I) molecule optimized at the HF/Jorge-TZP(DK) and MP2/Jorge-TZP(DK) levels of theory compared to X-ray diffraction experimental values. Averages over all symmetrically equivalent bonds are presented.

	X=F			X=Cl			X=Br			X=I		
	HF	MP2	Exp ^a^	HF	MP2	Exp ^b^	HF	MP2	Exp ^a^	HF	MP2	Exp ^a^
C-X	1.277	1.303	1.297	1.711	1.701	1.694	1.879	1.839	1.853	2.104	2.074	2.097
C-N	1.509	1.516	1.525	1.520	1.533	1.542	1.519	1.532	1.532	1.513	1.526	1.535
N-O_1_	1.171	1.219	1.206	1.171	1.217	1.210	1.171	1.217	1.198	1.172	1.217	1.207
N-O_2_	1.172	1.220	1.215	1.172	1.220	1.213	1.172	1.220	1.219	1.173	1.221	1.209
X-C-N	109.98	110.48	110.4	111.68	112.19	112.5	111.86	112.74	112.6	111.61	112.34	112.8
X-C-N-O_1_	−39.54	−40.20	−36.3	−43.31	−43.19	−42.2	−44.40	−44.74	−42.2	−45.67	−45.91	−44.8

a. X-ray diffraction from Ref [41]; b. X-Ray diffraction data from Ref [36].

**Table 2:** Atomic charges of the XC(NO_2_)_3_ compounds (X=F, Cl, Br, and I) extracted from the QUAO analysis.

	X=F	X=Cl	X=Br	X=I
C	0.566	0.203	0.136	0.08
X	−0.249	0.088	0.159	0.214
N	0.686	0.702	0.704	0.706
O_1_	−0.396	−0.401	−0.403	−0.405
O_2_	−0.396	−0.399	−0.399	−0.399

**Table 3:** Difference in KBO (ΔKBO) between the NH_3_--XC(NO_2_)_3_ complex and the XC(NO_2_)_3_ optimized compound in kcal/mol. The sum from all symmetrically equivalent bonds (for instance, all three Cnσ-Ncσ QUAO pairs) are included.

QUAO Pair	X=F	X=Cl	X=Br	X=I
Xcσ-Cxσ	−3.8	+3.6	+4.8	+6.9
Cnσ-Ncσ	+1.8	+1.0	−2.1	−13.2
O_1_nσ-No_1_σ	−0.9	+0.2	+1.4	+5.6
O_2_nσ-No_2_σ	+1.4	+1.6	+1.6	+2.4
O-N-O π bond	+0.3	+0.9	+1.5	+4.1
O_1_lp-NC ^a^	+1.4	+3.7	+5.6	+13.9
O_2_lp-NC ^a^	+1.6	+3.1	+3.6	+6.8
Xlp-NC ^a^	−6.1	−4.5	−3.3	+6.5

a. NC includes both the Ncσ and Cnσ QUAOs.

**Table 4:** Intermolecular forces (IMFs) between ammonia and the XC(NO_2_)_3_ molecule at the MP2/Def2-TZVP level of theory on the HF/Jorge-TZP(DK) optimized structure, in kcal/mol, without counter-poise correction and with counter-poise correction (in parentheses).

IMF	X=F	X=Cl	X=Br	X=I
Electrostatic	−1.20 (−1.29)	−6.64 (−6.80)	−10.55 (−10.59)	−27.32 (−26.00)
Polarization	−0.19 (−0.15)	−1.46 (−1.58)	−2.82 (−3.17)	−11.22 (−12.84)
Dispersion	−0.33 (−0.20)	−0.86 (−0.52)	−1.46 (−0.94)	−2.39 (−1.28)
Exchange	−0.38 (−0.59)	−5.74 (−5.87)	−10.59 (−10.78)	−35.73 (−35.88)
Repulsion	0.70 (1.05)	9.80 (10.39)	18.45 (19.40)	65.90 (67.00)
Δ*E_HF_*	−1.07 (−0.97)	−4.05 (−3.86)	−5.50 (−5.14)	−8.36 (−7.72)
Δ*E_MP_*_2_	−1.39 (−1.17)	−4.91 (−4.38)	−6.96 (−6.08)	−10.75 (−9.01)

## Data Availability

All the data are available in the paper and Supplementary Materials.

## Supplementary Materials

The following supporting information can be downloaded at: https://www.mdpi.com/article/10.3390/molecules30091986/s1, Table S1: Bond distances (Å), angles (°), interaction energies Δ*E* (in kcal/mol), and imaginary frequencies (in cm^−1^) of the NH_3_--XC(NO_2_)_3_ (X=F, Cl, Br, and I) molecule optimized at the HF/Jorge-TZP(DK) and MP2/Jorge-TZP(DK) (in parentheses) level of theory. Table S2: Difference in QUAO occupation between the NH_3_--XC(NO_2_)_3_ complex and the XC(NO_2_)_3_ optimized monomer. Table S3: Difference ΔKBO between KBOs in the XC(NO_2_)_3_ molecule at the dimer geometry and the optimized XC(NO_2_)_3_ molecule. Table S4: Decomposition of the total interaction energy between ammonia and the XC(NO_2_)_3_ molecule at the MP2/Def2-TZVP level of theory on the MP2/Jorge-TZP(DK) optimized structure. Tables S5–S8: Contributions of the electrons kinetic energy (T), electron–nucleus potential energy (V), electron–electron exchange energy (X), electron–electron repulsion energy (J), and nucleus–nucleus repulsion energy (N) in kcal/mol to the interaction energy terms for the NH_3_--XC(NO_2_)_3_ complexes optimized at the HF/Jorge-TZP(DK) level of theory. The coordinates of optimized systems are also given in the SI. Figure S1: Correlation between the kinetic energy (KE) contribution of the polarization energy and the total interaction energy and between the kinetic bond order (KBO) from the QUAO analysis and the total interaction energy. Figure S2. Decomposition of the total MP2 interaction energy between the NH_3_ molecule and the XC(NO_2_)_3_ compound as a function of the *R*_NX_ distance: (A) X=F, (B) X=Cl, (C) X=Br, and (D) X=I. Figure S3. Decomposition of the total MP2 interaction energy with CP correction between the NH_3_ molecule and the XC(NO_2_)_3_ compound as a function of the N--X distance: (A) X=F, (B) X=Cl, (C) X=Br, and (D) X=I. Figure S4. Molecular electrostatic potential maps of the NH_3_--XC(NO_2_)_3_ complexes (X=F, Cl, Br, and I) at θ_NXC_ = 180° (left panel) and θ_NXC_ = 136° (right panel). All optimized geometries are also available.
