# Supplementary material for: Decrypting the Unusual Structure and σ-Hole Interactions of the XC(NO2)3 (X=F, Cl, Br, and I) Compounds Using Quasi-Atomic Orbitals
Source: Molecules. 2025 Apr 29;30(9):1986. doi: 10.3390/molecules30091986 (PMC12073642; doi:10.3390/molecules30091986)
Supplement: Supplementary file 1 [file molecules-30-01986-s001.zip › molecules-3514167-supplementary.pdf]

Supporting Information for

Decrypting the unusual structure and σ-hole interactions of the $XC(NO_2)_3$ (X= F, Cl, Br, I) compounds using quasi-atomic orbitals.

.

Emilie B. Guidez*

**Department of chemistry, University of Colorado Denver, Denver CO 80204*

Table S1. Bond distances (Å), angles (°) of the NH_3--$XC(NO_2)_3$ (X=F, Cl, Br, I) complex optimized at the HF/Jorge-TZP(DK) and MP2/Jorge-TZP(DK) (in parentheses) level of theory.

	X=F	X=Cl	X=Br	X=I
C-X	1.273 (1.298)	1.710 (1.703)	1.885 (1.850)	2.149 (2.126)
C-N	1.509 (1.517)	1.520 (1.532)	1.518 (1.529)	1.507 (1.515)
N-O_1	1.171 (1.219)	1.171 (1.217)	1.172 (1.218)	1.174 (1.220)
N-O_2	1.172 (1.220)	1.173 (1.221)	1.173 (1.221)	1.174 (1.223)
N--X	3.395 (3.063)	3.062 (2.876)	2.972 (2.843)	2.686 (2.615)
X-C-N angle	110.16 (110.69)	111.91 (112.44)	112.09 (112.94)	111.65 (112.13)
ΔE (kcal/mol)	-1.05 (-1.54)	-3.98 (-5.27)	-5.51 (-7.07)	-10.56 (-13.19)
Imaginary frequencies	36.37 (3.86)	N/A (33.33)	52.96 (N/A)	N/A (N/A)

Table S2. Difference in QUAO occupation between the NH_3--$XC(NO_2)_3$ complex and the $XC(NO_2)_3$ optimized monomer. The sums from all symmetrically equivalent bonds (for instance all three Cnσ QUAOs) are included. The change in occupation of the bonds given in bold corresponds to the contribution of the two QUAOs forming the bond (in the two previous rows).

QUAO	X=F	X=Cl	X=Br	X=I
Xcσ	-0.006	-0.022	-0.030	-0.047
Cxσ	0.006	0.031	0.052	0.124
**C-X bond**	**0.000**	**0.009**	**0.022**	**0.077**
Cnσ	-0.009	-0.021	-0.031	-0.070
Ncσ	0.010	0.018	0.021	0.038
**C-N bond**	**0.001**	**-0.003**	**-0.010**	**-0.032**
O_1nσ	0.002	0.002	0.003	0.005
No_1σ	-0.002	-0.003	-0.003	-0.005
**O_1-N bond**	**0.000**	**-0.001**	**0.000**	**0.000**
O_2nσ	0.001	0.002	0.003	0.006
No_2σ	-0.001	-0.002	-0.002	-0.004
**O_2-N bond**	**0.000**	**0.000**	**0.001**	**0.002**
O_1lp	0.001	0.003	0.006	0.013
O_2lp	0.002	0.005	0.006	0.013
Xlp	-0.005	-0.005	-0.001	0.009

Table S3. Difference ΔKBO between KBOs in the $XC(NO_2)_3$ molecule at the dimer geometry and the optimized $XC(NO_2)_3$ molecule. All values are in kcal/mol.

	X=F	X=Cl	X=Br	X=I
Xcσ - Cxσ	-2.5	-0.4	+0.2	+1.5
Cnσ - Ncσ	+0.9	-0.1	-2.1	-10.4
O_1nσ - No_1σ	-0.6	+1.0	+2.4	+7.6
O_2nσ - No_2σ	+1.8	+2.3	+2.5	+3.9
O-N-O π bond	+0.7	+1.7	+2.4	+5.2
O_1lp - NC[a]	+0.5	+1.2	+3.0	+7.6
O_2lp - NC[a]	+0.1	-0.4	-0.6	-1.0
Xlp - NC[a]	-3.0	-0.8	-0.7	+8.0

a. NC includes both the Ncσ and Cnσ QUAOs

Table S4. Decomposition of the total interaction energy between NH_3 and the $XC(NO_2)_3$ molecule at the MP2/Def2-TZVP level of theory on the MP2/Jorge-TZP(DK) optimized structure, in kcal/mol, without counter-poise correction and with counter-poise correction (in parentheses).

IMF	X=F	X=Cl	X=Br	X=I
Electrostatic	-1.86 (-1.98)	-9.63 (-9.58)	-13.91 (-13.68)	-32.08 (-30.31)
Polarization	-0.38 (-0.30)	-2.41 (-2.64)	-3.93 (-4.46)	-13.76 (-15.59)
Dispersion	-0.60 (-0.35)	-1.21 (-0.74)	-1.73 (-1.08)	-2.37 (-1.16)
Exchange	-1.45 (-1.72)	-10.30 (-10.07)	-15.43 (-15.34)	-43.03 (-42.86)
Repulsion	2.64 (3.12)	18.07 (18.28)	27.55 (28.19)	80.54 (81.10)
ΔE_{HF}	-1.06 (-0.88)	-4.27 (-4.01)	-5.72 (-5.29)	-8.33 (-7.66)
ΔE_{MP2}	-1.65 (-1.23)	-5.48 (-4.74)	-7.44 (-6.37)	-10.70 (-8.82)

Table S5. Contributions of the electrons kinetic energy (T), electron-nucleus potential energy (V), electron-electron exchange energy (X), electron-electron repulsion energy (J) and nucleus-nucleus repulsion energy (N) in kcal/mol to the interaction energy terms for the NH_3--$FC(NO_2)_3$ complex optimized at the HF/Jorge-TZP(DK) level of theory. No counter-poise correction is included.

X= F	T	V	X	J	N	V+X+J+N
Electrostatic	0.00	-106161.40	0.00	53289.74	52870.46	-1.20
Exchange	0.00	0.00	-0.38	0.00	0.00	-0.38
Repulsion	3.59	-5.60	-0.15	2.86	0.00	-2.88
Polarization	-4.21	16.69	6.81	-19.48	0.00	4.02
Total	-0.63	11.09	6.28	-16.62	0.00	-0.44

Table S6. Contributions of the electrons kinetic energy (T), electron-nucleus potential energy (V), electron-electron exchange energy (X), electron-electron repulsion energy (J) and nucleus-nucleus repulsion energy (N) in kcal/mol to the interaction energy terms for the NH_3--$ClC(NO_2)_3$ complex optimized at the HF/Jorge-TZP(DK) level of theory. No counter-poise correction is included.

	T	V	X	J	N	V+X+J+N
Electrostatic	0.00	-122123.71	0.00	61297.55	60819.53	-6.64
Exchange	0.00	0.00	-5.74	0.00	0.00	-5.74
Repulsion	42.78	-79.27	-0.06	46.35	0.00	-32.98
Polarization	-32.59	-22.93	-31.60	85.65	0.00	31.13
Total	10.19	-102.20	-37.40	132.00	0.00	-14.24

Table S7. Contributions of the electrons kinetic energy (T), electron-nucleus potential energy (V), electron-electron exchange energy (X), electron-electron repulsion energy (J) and nucleus-nucleus repulsion energy (N) in kcal/mol to the interaction energy terms for the NH_3--$BrC(NO_2)_3$ complex optimized at the HF/Jorge-TZP(DK) level of theory. No counter-poise correction is included.

	T	V	X	J	N	V+X+J+N
Electrostatic	0.00	-160703.84	0.00	80737.33	79955.96	-10.55
Exchange	0.00	0.00	-10.59	0.00	0.00	-10.59
Repulsion	80.07	-156.64	0.50	94.53	0.00	-61.62
Polarization	-62.19	41.19	4.25	13.93	0.00	59.38
Total	17.88	-115.45	-5.84	108.46	0.00	-23.38

Table S8. Contributions of the electrons kinetic energy (T), electron-nucleus potential energy (V), electron-electron exchange energy (X), electron-electron repulsion energy (J) and nucleus-nucleus repulsion energy (N) in kcal/mol to the interaction energy terms for the NH_3--$IC(NO_2)_3$ complex optimized at the HF/Jorge-TZP(DK) level of theory. No counter-poise correction is included.

	T	V	X	J	N	V+X+J+N
Electrostatic	0.00	-145079.33	0.00	72809.97	72242.04	-27.32
Exchange	0.00	0.00	-35.73	0.00	0.00	-35.73
Repulsion	166.89	-285.47	8.79	175.70	0.00	-100.99
Polarization	-89.45	-222.55	-261.70	562.48	0.00	78.23
Total	77.44	-508.03	-288.64	738.18	0.00	-85.80

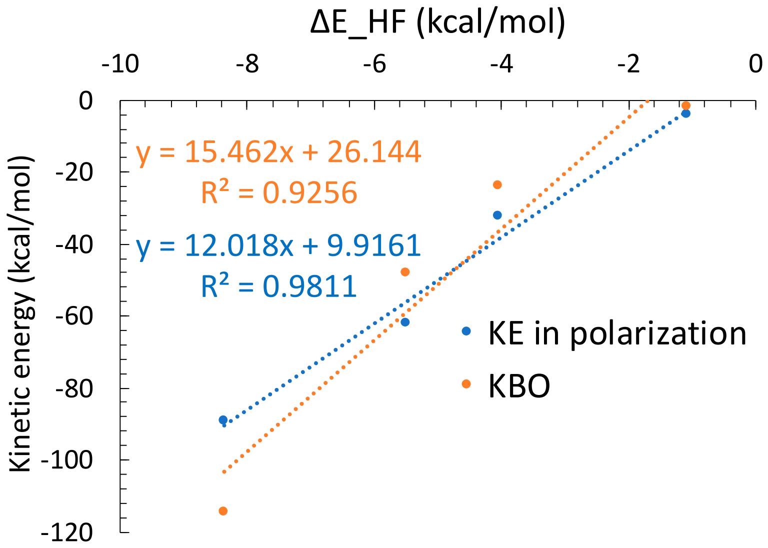

Figure S1. Correlation between A) the kinetic energy (KE) contribution of the polarization energy and the total interaction energy (blue) B) the kinetic bond order (KBO) from the QUAO analysis and the total interaction energy (orange). Each point corresponds to a different NH_3---$XC(NO_2)_3$ (X= F, Cl, Br, I) halogenated system.

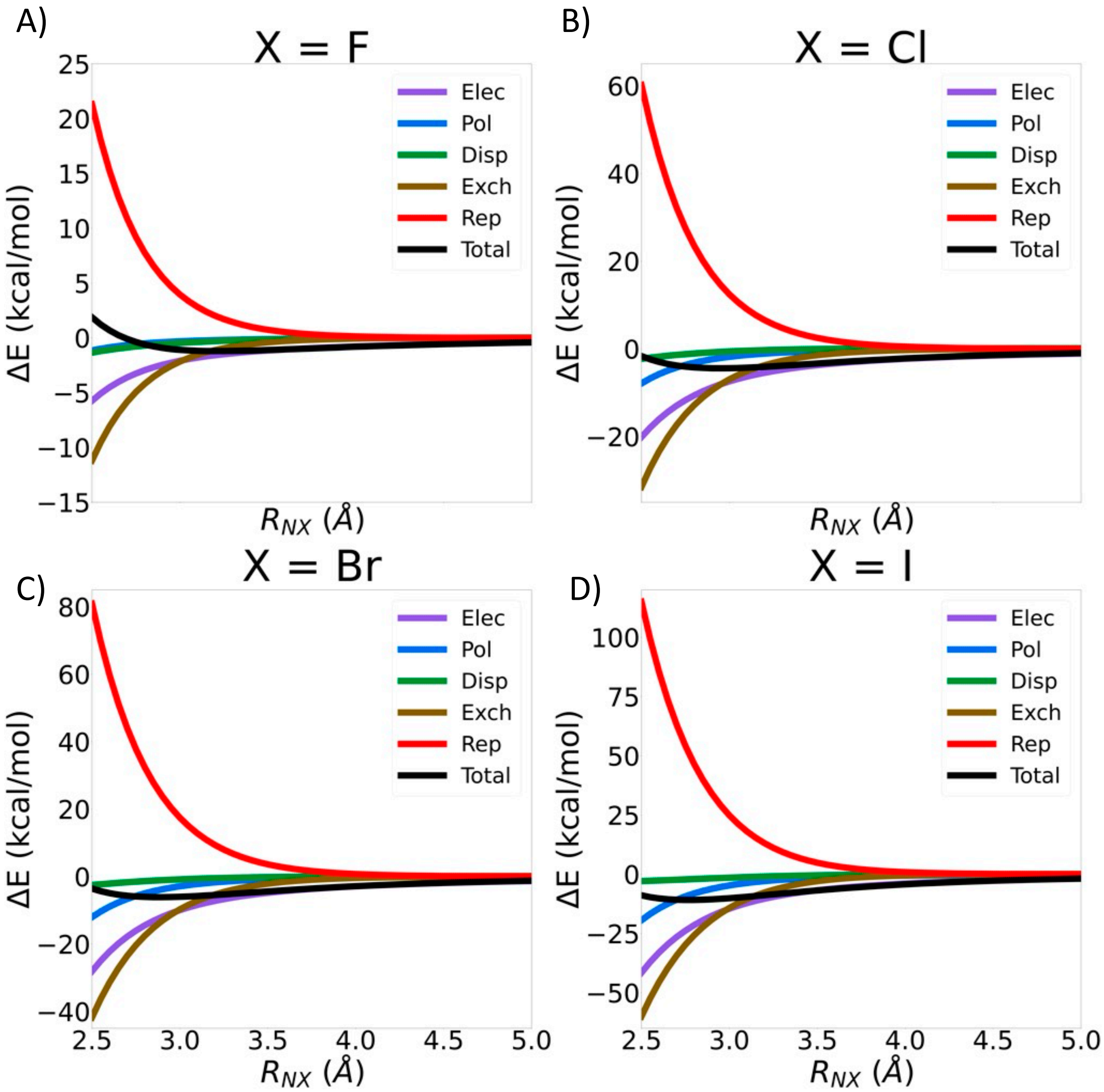

Figure S2. Decomposition of the total MP2 interaction energy between the NH_3 molecule and the $XC(NO_2)_3$ compound as a function of the R_{NX} distance. A) X=F B) X=Cl C) X=Br and D) X=I.

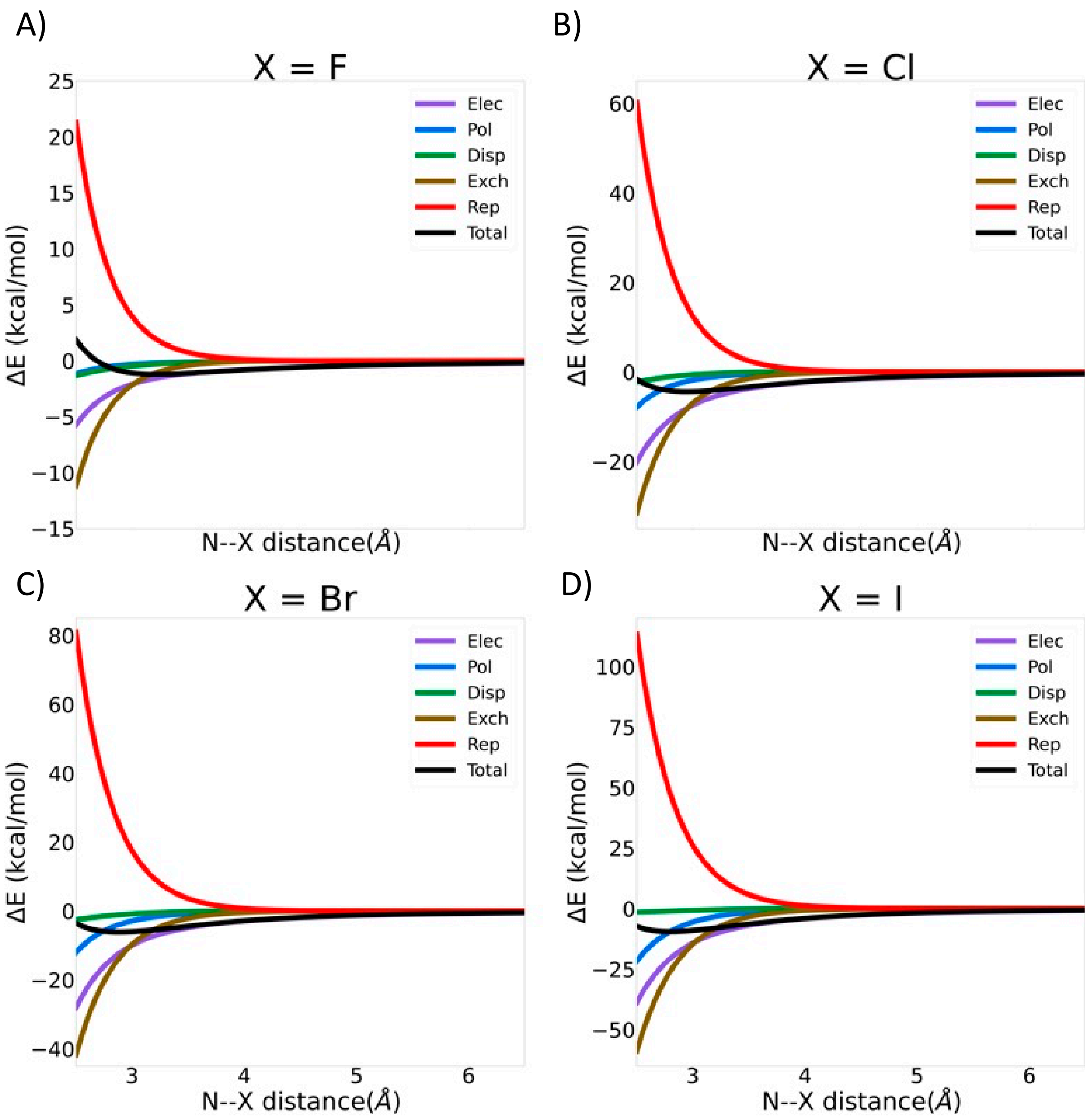

Figure S3. Decomposition of the total MP2 interaction energy with CP correction between the NH_3 molecule and the $XC(NO_2)_3$ compound as a function of the N--X distance. A) X=F B) X=Cl C) X=Br and D) X=I

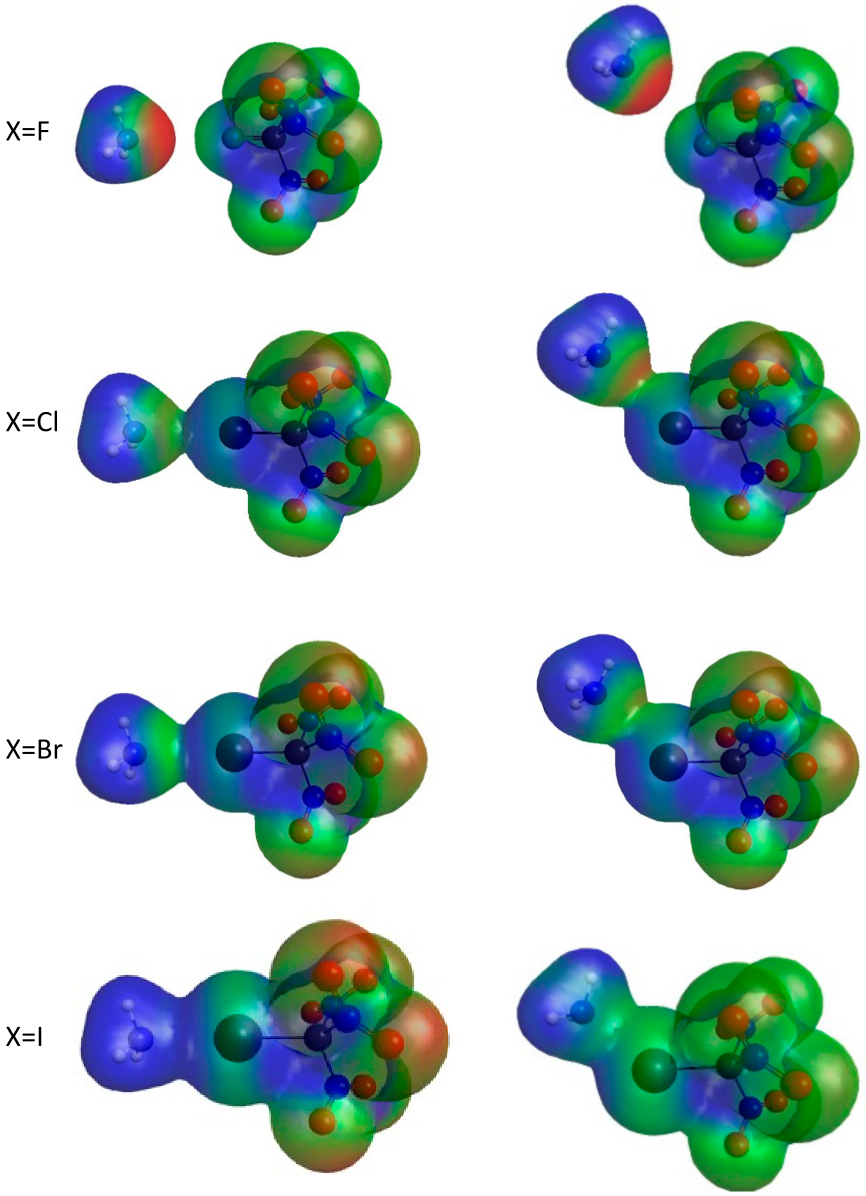

Figure S4. Molecular electrostatic potential maps of the NH_3--$XC(NO_2)_3$ complexes (X= F, Cl, Br, I) at θ_{NXC}=180° (left panel) and θ_{NXC}=136° (right panel).

Optimized $FC(NO_2)_3$ at the MP2/Jorge-TZP with Douglas-Kroll Hamiltonian.

C	-0.0000000000	0.0000000000	-0.4035150968
F	-0.0000000000	0.0000000000	-1.7061935759
N	-0.4931813879	-1.3319592398	0.1270158551
N	1.4001012324	0.2388720093	0.1270158551
N	-0.9069198445	1.0930872305	0.1270158551
O	-1.4320056650	-1.7987345056	-0.4950689910
O	2.2737526090	-0.3407860314	-0.4950689910
O	-0.8417469440	2.1395205370	-0.4950689910
O	0.0783375241	-1.7688468602	1.1123765402
O	1.4926975543	0.9522657160	1.1123765402
O	-1.5710350784	0.8165811441	1.1123765402

Optimized $ClC(NO_2)_3$ at the MP2/Jorge-TZP with Douglas-Kroll Hamiltonian.

C	0.0000000000	-0.0000000000	-0.4214634339
CL	0.0000000000	0.0000000000	-2.1228419717
N	-0.4953738289	-1.3301924144	0.1576457777
N	1.3996673372	0.2360898870	0.1576457777
N	-0.9042935083	1.0941025274	0.1576457777
O	-1.4746522367	-1.7998636830	-0.3905390081
O	2.2960537912	-0.3771544572	-0.3905390081
O	-0.8214015545	2.1770181402	-0.3905390081
O	0.1212641445	-1.7520052926	1.1220822123
O	1.4566490187	0.9810204761	1.1220822123
O	-1.5779131632	0.7709848166	1.1220822123

Optimized $BrC(NO_2)_3$ at the MP2/Jorge-TZP with Douglas-Kroll Hamiltonian.

C	0.0000000000	0.0000000000	-0.4238254444
BR	-0.0000000000	0.0000000000	-2.2630350183
N	-0.4901707378	-1.3247173057	0.1683108110
N	1.3923242084	0.2378583418	0.1683108110
N	-0.9021534707	1.0868589639	0.1683108110
O	-1.4879547384	-1.7887805672	-0.3517739577
O	2.2931067822	-0.3942163195	-0.3517739577
O	-0.8051520438	2.1829968867	-0.3517739577
O	0.1421483867	-1.7530643928	1.1201704809
O	1.4471241053	0.9996363104	1.1201704809
O	-1.5892724920	0.7534280824	1.1201704809

Optimized $IC(NO_2)_3$ at the MP2/Jorge-TZP with Douglas-Kroll Hamiltonian.

C	0.0000000000	0.0000000000	-0.3931032430
I	0.0000000000	-0.0000000000	-2.4666734571
N	-0.4861756259	-1.3246503959	0.1866439464

N	1.3902687069	0.2412847552	0.1866439464
N	-0.9040930810	1.0833656407	0.1866439464
O	-1.4939177128	-1.7786327185	-0.3227035039
O	2.2872999746	-0.4044543312	-0.3227035039
O	-0.7933822618	2.1830870497	-0.3227035039
O	0.1536939096	-1.7612001133	1.1304056375
O	1.4483970845	1.0137028868	1.1304056375
O	-1.6020909941	0.7474972266	1.1304056375

Optimized NH_3---$FC(NO_2)_3$ at the MP2/Jorge-TZP with Douglas-Kroll Hamiltonian.

C	-0.0000000000	-0.0000000000	-0.5228231221
F	-0.0000000000	-0.0000000000	-1.8203420635
N	1.3987228314	0.2423406525	0.0133165212
N	-0.9092345771	1.0901591786	0.0133165212
N	-0.4894882543	-1.3324998311	0.0133165212
O	2.2762601469	-0.3385859363	-0.6022269043
O	-0.8449060512	2.1405920810	-0.6022269043
O	-1.4313540957	-1.8020061447	-0.6022269043
O	1.4891881591	0.9610740379	0.9954679113
O	-1.5769086113	0.8091377578	0.9954679113
O	0.0877204522	-1.7702117958	0.9954679113
N	-0.0000000000	-0.0000000000	-4.8835582779
H	-0.9288621535	0.1126209852	-5.2726143238
H	0.3668984426	-0.8607287142	-5.2726143238
H	0.5619637110	0.7481077290	-5.2726143238

Optimized NH_3---$ClC(NO_2)_3$ at the MP2/Jorge-TZP with Douglas-Kroll Hamiltonian.

C	0.0000000000	0.0000000000	-0.1070808554
CL	0.0000000000	0.0000000000	-1.8102564896
N	1.3950027721	0.2417331043	0.4777151870
N	-0.9068483953	1.0872412868	0.4777151870
N	-0.4881543768	-1.3289743911	0.4777151870
O	2.2962097150	-0.3735642457	-0.0618319480
O	-0.8245887308	2.1753580685	-0.0618319480
O	-1.4716209842	-1.8017938228	-0.0618319480
O	1.4530286630	0.9955369548	1.4362768496
O	-1.5886746247	0.7605912572	1.4362768496
O	0.1356459617	-1.7561282120	1.4362768496
N	0.0000000000	0.0000000000	-4.6865808362
H	0.3009693798	-0.8874379452	-5.0728545983
H	0.6180591149	0.7043661013	-5.0728545983
H	-0.9190284947	0.1830718439	-5.0728545983

Optimized NH_3---$BrC(NO_2)_3$ at the MP2/Jorge-TZP with Douglas-Kroll Hamiltonian.

C	0.0000000000	0.0000000000	-0.1300104089

BR	0.0000000000	0.0000000000	-1.9804513110
N	1.3843801130	0.2554157851	0.4656629251
N	-0.9133866149	1.0712004538	0.4656629251
N	-0.4709934981	-1.3266162389	0.4656629251
O	2.2944792993	-0.3719695946	-0.0462223046
O	-0.8251045313	2.1730621589	-0.0462223046
O	-1.4693747680	-1.8010925643	-0.0462223046
O	1.4368709234	1.0274712898	1.4107099908
O	-1.6082517003	0.7306310767	1.4107099908
O	0.1713807769	-1.7581023665	1.4107099908
N	0.0000000000	0.0000000000	-4.8239038860
H	0.2091313818	-0.9146648065	-5.2073626460
H	0.6875572675	0.6384454926	-5.2073626460
H	-0.8966886493	0.2762193139	-5.2073626460

Optimized NH_3---$IC(NO_2)_3$ at the MP2/Jorge-TZP with Douglas-Kroll Hamiltonian.

C	0.0000000000	0.0000000000	-0.2853894317
I	0.0000000000	0.0000000000	-2.4109556761
N	1.3822856199	0.2413029669	0.2851859078
N	-0.9001173093	1.0764429787	0.2851859078
N	-0.4821683106	-1.3177459456	0.2851859078
O	2.2730485842	-0.4256980551	-0.2138115068
O	-0.7678589621	2.1813668455	-0.2138115068
O	-1.5051896221	-1.7556687904	-0.2138115068
O	1.4698210412	1.0403051052	1.2070190137
O	-1.6358411694	0.7527498081	1.2070190137
O	0.1660201282	-1.7930549133	1.2070190137
N	0.0000000000	0.0000000000	-5.0262466622
H	0.3833496268	-0.8629655200	-5.3928633947
H	0.5556752495	0.7634732754	-5.3928633947
H	-0.9390248763	0.0994922446	-5.3928633947

Optimized $FC(NO_2)_3$ at the HF/Jorge-TZP with Douglas-Kroll Hamiltonian.

C	-0.0000000000	0.0000000000	-0.3843887296
F	-0.0000000000	0.0000000000	-1.6610801486
N	-0.4954068508	-1.3286285678	0.1312682486
N	1.3983295173	0.2352793659	0.1312682486
N	-0.9029226665	1.0933492019	0.1312682486
O	-1.3886695902	-1.7758643758	-0.4804380398
O	2.2322784582	-0.3146909547	-0.4804380398
O	-0.8436088680	2.0905553305	-0.4804380398
O	0.0552054483	-1.7582744175	1.0720799307
O	1.4951075882	0.9269465294	1.0720799307
O	-1.5503130365	0.8313278881	1.0720799307

Optimized $ClC(NO_2)_3$ at the HF/Jorge-TZP with Douglas-Kroll Hamiltonian.

C	-0.0000000000	0.0000000000	-0.3964254386
CL	-0.0000000000	-0.0000000000	-2.1072058860
N	-0.4953364014	-1.3230410603	0.1652323907
N	1.3934553691	0.2325466231	0.1652323907
N	-0.8981189678	1.0904944371	0.1652323907
O	-1.4378437613	-1.7703211838	-0.3655764462
O	2.2520649987	-0.3600486321	-0.3655764462
O	-0.8142212374	2.1303698159	-0.3655764462
O	0.1084004855	-1.7470615902	1.0759750105
O	1.4587994763	0.9674083693	1.0759750105
O	-1.5671999618	0.7796532208	1.0759750105

Optimized $BrC(NO_2)_3$ at the HF/Jorge-TZP with Douglas-Kroll Hamiltonian.

C	0.0000000000	0.0000000000	-0.3863780998
BR	-0.0000000000	-0.0000000000	-2.2651744436
N	-0.4925329032	-1.3206899571	0.1791606545
N	1.3900175049	0.2337989722	0.1791606545
N	-0.8974846018	1.0868909849	0.1791606545
O	-1.4471258450	-1.7635821396	-0.3343936761
O	2.2508698570	-0.3714566744	-0.3343936761
O	-0.8037440121	2.1350388140	-0.3343936761
O	0.1221495747	-1.7507068814	1.0801710495
O	1.4550818465	0.9811380755	1.0801710495
O	-1.5772314212	0.7695688059	1.0801710495

Optimized $IC(NO_2)_3$ at the HF/Jorge-TZP with Douglas-Kroll Hamiltonian.

C	-0.0000000000	0.0000000000	-0.3591632570
I	-0.0000000000	0.0000000000	-2.4635517361
N	-0.4871773907	-1.3193886758	0.1979156625
N	1.3862128060	0.2377863414	0.1979156625
N	-0.8990354153	1.0816023344	0.1979156625
O	-1.4534186791	-1.7522067719	-0.3035500443
O	2.2441649167	-0.3825941125	-0.3035500443
O	-0.7907462376	2.1348008844	-0.3035500443
O	0.1358636947	-1.7614359799	1.0876265595
O	1.4575164584	0.9983794010	1.0876265595
O	-1.5933801530	0.7630565789	1.0876265595

Optimized NH_3---$FC(NO_2)_3$ at the HF/Jorge-TZP with Douglas-Kroll Hamiltonian.

C	0.0000000000	0.0000000000	-0.4321988762
F	0.0000000000	-0.0000000000	-1.7049368419
N	1.3966718157	0.2390666192	0.0880672795
N	-0.9053736733	1.0900199636	0.0880672795
N	-0.4912981425	-1.3290865828	0.0880672795

O	2.2343119222	-0.3131209141	-0.5161818461
O	-0.8459852950	2.0915313417	-0.5161818461
O	-1.3883266272	-1.7784104276	-0.5161818461
O	1.4903738427	0.9362676137	1.0259546573
O	-1.5560184595	0.8225678021	1.0259546573
O	0.0656446168	-1.7588354157	1.0259546573
N	0.0000000000	-0.0000000000	-5.1001040213
H	-0.9259142261	0.1131935960	-5.4603914609
H	0.3649285834	-0.8584620395	-5.4603914609
H	0.5609856427	0.7452684436	-5.4603914609

Optimized NH_3---$ClC(NO_2)_3$ at the HF/Jorge-TZP with Douglas-Kroll Hamiltonian.

C	-0.0000000000	-0.0000000000	-0.0417790332
CL	-0.0000000000	-0.0000000000	-1.7521499746
N	1.3925607631	0.2240584448	0.5252971331
N	-0.8903206867	1.0939637747	0.5252971331
N	-0.5022400764	-1.3180222195	0.5252971331
O	2.2500624342	-0.3769180812	0.0014309325
O	-0.7986105836	2.1370702688	0.0014309325
O	-1.4514518506	-1.7601521875	0.0014309325
O	1.4639394197	0.9636568868	1.4329334372
O	-1.5665210544	0.7859802836	1.4329334372
O	0.1025816347	-1.7496371705	1.4329334372
N	-0.0000000000	-0.0000000000	-4.8157755768
H	0.4047055038	-0.8399920624	-5.1784272112
H	0.5251017131	0.7704812786	-5.1784272112
H	-0.9298072169	0.0695107838	-5.1784272112

Optimized NH_3---$BrC(NO_2)_3$ at the HF/Jorge-TZP with Douglas-Kroll Hamiltonian.

C	-0.0000000000	0.0000000000	-0.0648589794
BR	-0.0000000000	0.0000000000	-1.9502051837
N	1.3843524652	0.2469452535	0.5057363514
N	-0.9060370954	1.0754117759	0.5057363514
N	-0.4783153698	-1.3223570294	0.5057363514
O	2.2530909022	-0.3543611413	-0.0009797018
O	-0.8196597006	2.1284145289	-0.0009797018
O	-1.4334312015	-1.7740533877	-0.0009797018
O	1.4466195355	1.0010244368	1.4026721402
O	-1.5902223598	0.7522970490	1.4026721402
O	0.1436028243	-1.7533214857	1.4026721402
N	-0.0000000000	0.0000000000	-4.9217011589
H	0.2464463240	-0.8994235822	-5.2838409191
H	0.6557005089	0.6631405683	-5.2838409191
H	-0.9021468329	0.2362830139	-5.2838409191

Optimized NH_3---$IC(NO_2)_3$ at the HF/Jorge-TZP with Douglas-Kroll Hamiltonian.

C	0.0000000000	-0.0000000000	-0.2447686140
I	-0.0000000000	0.0000000000	-2.3937216842
N	1.3822457191	0.2293838836	0.3113302357
N	-0.8897751300	1.0823679652	0.3113302357
N	-0.4924705891	-1.3117518488	0.3113302357
O	2.2346160568	-0.4084507811	-0.1834512942
O	-0.7635792757	2.1394596634	-0.1834512942
O	-1.4710367810	-1.7310088823	-0.1834512942
O	1.4779663860	1.0048493170	1.1881164357
O	-1.6092082285	0.7775317778	1.1881164357
O	0.1312418425	-1.7823810947	1.1881164357
N	-0.0000000000	0.0000000000	-5.0795358253
H	0.4263744318	-0.8329639606	-5.4319872393
H	0.5081807344	0.7857330698	-5.4319872393
H	-0.9345551662	0.0472308908	-5.4319872393
